# A distal enhancer with ETV4 binding is critical for UCP1 expression and thermogenesis in brown fat

**DOI:** 10.1101/gad.352748.125

**Published:** 2025-07-01

**Authors:** Pengya Xue, Nicholas Holloway, Alexander Tran, Frances Lin, Jennie Dinh, Caleb Yang, Yuhui Wang, Danielle Yi, Hei Sook Sul

**Affiliations:** 1 Department of Nutritional Sciences and Toxicology, University of California, Berkeley, Berkeley, California 94720, USA;; 2 Endocrinology Program, University of California, Berkeley, Berkeley, California 94720, USA

**Keywords:** UCP1, brown fat, thermogenesis, transcription factor

## Abstract

Brown adipose tissue (BAT) dissipates energy as heat in maintaining body temperature, and BAT mass inversely correlates with adiposity. During thermogenesis, BAT generates heat by uncoupling respiration through UCP1, and the −2.5 kb enhancer of *UCP1* gene is known to activate *UCP1* expression upon cold or β-adrenergic stimulation. Here, we identify a critical *UCP1* enhancer located at 12 kb upstream of the *UCP1* gene locus that functions through chromatin looping and uncover its essential role in *UCP1* activation and thermogenesis by CRISPR activation and CRISPR interference in mice. Importantly, we identify ETV4 as a key regulator of *UCP1* expression by binding the −12 kb enhancer to promote chromatin accessibility and histone acetylation. Using gain- and loss-of-function approaches, we reveal that ETV4 enhances uncoupled respiration and thermogenesis, thereby protecting mice from diet-induced obesity and insulin resistance. The −12 kb enhancer and ETV4 can be potential therapeutic targets for combating obesity and improving metabolic health.

## Abstract (precis)

In this study, Xue et al. determine that a distal enhancer and its interaction with transcription factor ETV4 modulate the chromatin conformation and gene expression of UCP1, a key regulator of thermogenesis in brown adipose tissue. The work highlights distinct mechanisms sustaining body temperature and energy expenditure that can be leveraged to protect against metabolic diseases like diet-induced obesity and insulin resistance.

Obesity, characterized by excessive white adipose tissue (WAT), has escalated into a global health crisis, contributing significantly to metabolic disorders such as insulin resistance. While WAT primarily serves as an energy reservoir by storing triglycerides, brown adipose tissue (BAT) plays a crucial role in energy expenditure through nonshivering thermogenesis. The presence of BAT or BAT-like tissues in adult humans and the induction of BAT activity in response to cold exposure have been well documented (Cypess et al. 2009; Virtanen et al. 2009). Notably, BAT content is inversely correlated with body mass index and exhibits a robust capacity for glucose uptake, which aids in enhancing insulin sensitivity (van der Lans et al. 2013). A deeper understanding of the molecular mechanisms governing BAT function could reveal novel therapeutic targets for managing obesity and diabetes.

A key protein for thermogenesis in BAT is uncoupling protein 1 (UCP1), which is almost exclusively expressed in brown adipocytes. UCP1 is essential for nonshivering thermogenesis by dissipating the proton gradient across the inner mitochondrial membrane, uncoupling substrate oxidation from ATP synthesis and producing heat (Cannon and Nedergaard 2004). In response to cold exposure, sympathetic nerve stimulation releases norepinephrine, which activates the β3-adrenergic–cAMP–PKA signaling cascade, followed by transcriptional activation of thermogenic genes, including UCP1 (Robidoux et al. 2005). Various transcription factors are known to be recruited to the UCP1 upstream regulatory element to activate its transcription. The well-documented regulatory region of *UCP1* gene is the −2.5 kb region, which is known to be bound by transcription factors CREB/ATF4 and PPARγ/PGC1α and is critical and sufficient for transcription activation of UCP1 upon β3-adrenergic–cAMP–PKA signaling (Tontonoz et al. 1994; Cassard-Doulcier et al. 1998; Rim and Kozak 2002; Cao et al. 2004). Moreover, we identified the −5.5 kb and −70 bp regions that are bound by Zfp516 and Zc3h10, respectively, and are also involved in the activation of *UCP1* gene upon cold exposure or β3-adrenergic stimulation (Dempersmier et al. 2015; Yi et al. 2019, 2020).

As part of the multilayers of the transcriptional regulatory network, genome architecture and chromatin status play critical roles in determining the access of transcription factors to their target sites. Many epigenetic enzymes were identified to regulate UCP1 expression directly or indirectly; for example, HDAC3 and SUV420H2 (Emmett et al. 2017; Cui et al. 2024). Enhancers are pivotal DNA elements that regulate gene expression networks, defining cellular identity and responding to environmental signals by activating gene transcription (Heinz et al. 2015). Active enhancers are often found in nucleosome-depleted regions, with enriched specific epigenetic marks such as H3K4me1 and H3K27ac allowing them to interact with other regulatory elements and the transcriptional machinery by looping through the three-dimensional structure of the genome to interact with promoters of target genes (Kim and Wysocka 2023). Although the precise role is unclear, enhancers are also transcribed to produce enhancer RNAs (eRNAs), and their expression is often associated with active enhancers for gene transcription (Li et al. 2013). As a critical driver of thermogenesis, UCP1 is regulated mainly at the transcriptional level, and thus the multiple layers of UCP1 regulation bear important relevance to understanding the activation of thermogenesis and its influence on metabolic diseases.

Here, by using high-throughput sequencing technologies and genome-editing tools, we identified a critical enhancer located at −12 kb of the UCP1 transcription start site (TSS) that regulates *UCP1* activation. We further identified the transcription factor ETV4 as a key regulator that binds this enhancer, modulates chromatin accessibility, and drives UCP1 expression, thereby promoting thermogenesis and metabolic protection against obesity and diabetes.

## Results

### The presence of an active enhancer at the −12 kb region of the Ucp1 locus

**Figure 1.**
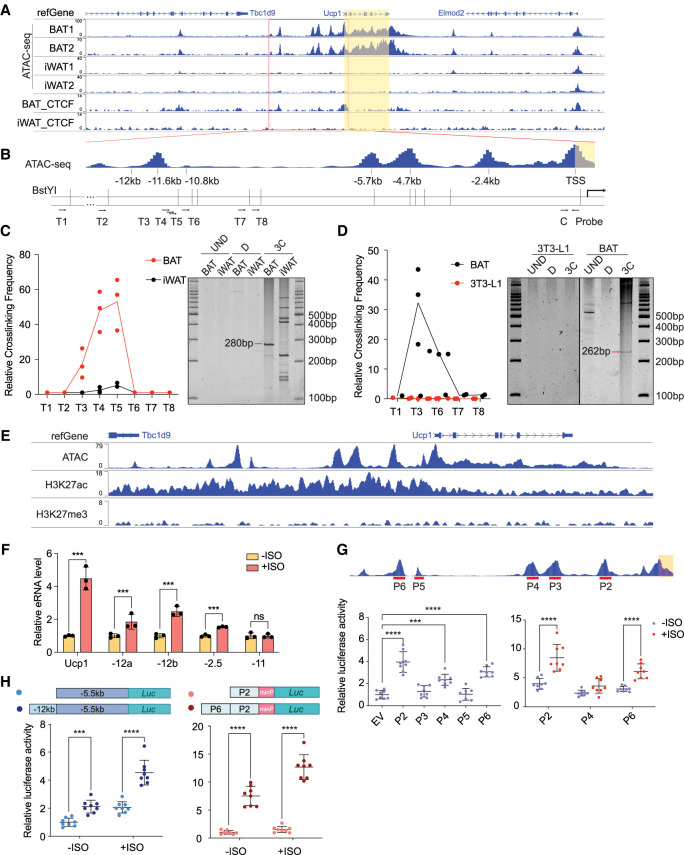
Interaction between the *Ucp1* −12 kb enhancer and the *Ucp1* proximal promoter. (*A*) ATAC-seq and CTCF CUT&RUN on *Ucp1* genome locus in brown and white adipose tissues (BAT and iWAT) showing the chromatin accessibility on the Ucp1 regulatory region. (*B*) Location of the chromatin opening on the *Ucp1* upstream genome locus and corresponding BstY1 restriction site. Arrows indicate the 3C primer and probe targets and directions. (T1-–T8) Testing primers, (C) Constant primer, (Probe) TaqMan probe. (*C*, *left*) 3C-qPCR showing the relative cross-linking frequency of the *Ucp1* −12 kb upstream locus and the *Ucp1* proximal promoter in BAT and iWAT. (*Right*) 3C PCR products visualized by TBE-PAGE gel showing the 280 bp cross-linked product in BAT and iWAT. (UND) Undigested genome DNA as PCR template, (D) digested but not ligated genome DNA as PCR template, (3C) 3C genome DNA as PCR template. (*D*, *left*) 3C-qPCR showing the relative cross-linking frequency of the *Ucp1* −12 kb upstream locus and the *Ucp1* proximal promoter in differentiated BAT cells and 3T3-L1 cells. (*Right*) 3C PCR products visualized by TBE-PAGE gel showing the 262 bp cross-linked product in differentiated BAT cells. (*E*) Genome browser view of ATAC-seq and H3K27ac and H3K27me3 CUT&RUN on the *Ucp1* upstream genome locus. (*F*) *Ucp1* RNA and upstream enhancer RNA levels in brown adipocytes upon isoproterenol (ISO) treatment (*n* = 3). (*G*, *top*) Measurement of the individual peak-driven luciferase activities. P2–P6 indicate the individual peaks upstream of the *Ucp1* genome locus. (*Bottom left*) Luciferase activity of the individual peaks measured. (*Bottom right*) Luciferase activities of P2, P4, and P6 were measured with or without isoproterenol (ISO) treatment (*n* = 8). (*H*, *top*) Constructs of the recombinant Ucp1 −12 kb region and other indicated region-driven luciferase reporters. (*Bottom*) Luciferase activity measured with or without ISO treatment (*n* = 8). (***) *P* < 0.005, (****) *P* < 0.001, (ns) nonsignificant based on two-way (*F*–*H*, *right*) or one-way ANOVA (*G*, *left*). All error bars represent the mean ± SEM.

To understand the chromatin status of the *Ucp1* genome locus that may control *Ucp1* transcription, we performed ATAC-seq to compare chromatin accessibility of the *Ucp1* locus in BAT and WAT. We detected an open chromatin region at −12 kb upstream of the *Ucp1* transcription start site (TSS) located just downstream from the neighboring gene locus, in addition to the −5.7 to −4.7 kb, −2.5 kb, and proximal promoter regions. Importantly, all of these open chromatin regions at the *Ucp1* genome locus were only detected in BAT but not in WAT (Fig. 1A). Because the −2.5 kb region has been well documented (Boyer and Kozak 1991; Kozak et al. 1994) and we previously reported the importance of the −4.5 to −5.5 kb upstream *cis*-element and −70 bp proximal promoter region for *Ucp1* expression, we chose to study the previously uncharacterized −12 kb region to explore whether this region functions through long-distance interaction to control *Ucp1* transcription. We first performed CUT&RUN in BAT to compare with WAT using an antibody against CTCF, which is known to function in forming chromatin loops (Mach et al. 2022). We detected CTCF binding at both the −12 kb region and the proximal promoter region of the *Ucp1* gene in BAT but not in WAT (Fig. 1A). Next, to investigate the potential interaction between the −12 kb region and the *Ucp1* promoter in vivo, we performed 3C in adipocytes isolated from BAT and WAT. Eight test primers (T1–T8) targeting the −12 kb region and nearby regions, as well as a constant primer (C) targeting the *Ucp1* proximal promoter region (−18 bp), were used in 3C experiments (Fig. 1B). TaqMan qPCR following 3C showed a strong interaction between the −12 kb region and the proximal promoter in brown adipocytes by using primers T3, T4, and T5, which target the −12 kb region, but not primers T1, T2, T6, T7, and T8, which target adjacent regions. However, those interactions were barely detectable in WAT (Fig. 1A, left). 3C products with expected sizes were visualized on the gel (Fig. 1C, right) and confirmed by Sanger sequencing (Supplemental Fig. S1A). In addition, we performed 3C in differentiated immortalized BAT cells to compare with 3T3-L1 adipocytes in culture. TaqMan qPCR following 3C detected a strong interaction between the −12 kb region and the proximal promoter only in differentiated BAT cells but not in differentiated 3T3-L1 cells (Fig. 1D, left). Again, the 3C products with expected sizes were visualized on PAGE gels (Fig. 1D, right) and confirmed by Sanger sequencing (Supplemental Fig. S1B). A second pair of primers was also used to confirm the 3C product (Supplemental Fig. S1C,D). Overall, these results demonstrate that the −12 kb *Ucp1* chromatin opening region interacts with the *Ucp1* proximal promoter through chromatin looping in brown adipocytes.

Next, to investigate whether the −12 kb peak of the *Ucp1* gene functions as an active enhancer, we examined histone marks H3K27ac and H3K27me3 in brown adipose tissue. CUT&RUN showed high enrichment of H3K27ac throughout the *Ucp1* genome locus including the −12 kb peak, whereas the repressive mark H3K27me3 was very low (Fig. 1E). Analysis of published ChIP-seq data sets (GSE63965) showed enrichment of H3K4me1 in the −12 kb region in BAT (Supplemental Fig. S1E; Harms et al. 2015). Enrichment of active histone marks H3K27ac and H3K4me1 and low enrichment of the repressive histone mark H3K27me3 further indicate the presence of an active enhancer at the −12 kb of the *Ucp1* genome locus.

Because enhancer regions are known to produce eRNA, we next examined potential eRNA expression in differentiated brown adipocytes using primers targeting the −12 kb peak region. Indeed, we detected eRNA produced from this region, whereas there was no eRNA generated from the −11 kb control region (Fig. 1F). Because *Ucp1* expression can be induced by the adrenergic agonist isoproterenol (ISO), we also measured eRNA levels in brown adipocytes treated with ISO. Although *Ucp1* level was increased by fourfold, eRNA produced from the −12 kb region was increased by twofold, as shown by qPCR using two different pairs of primers targeting this region. We also detected an increase in eRNA expression at the −2.5 kb region upon ISO treatment. These data further support the notion that the −12 kb *Ucp1* genome region is an active enhancer. In this regard, a published GRO-seq using BAT (GSE83928) also showed a bidirectional transcription of eRNA, as well as binding of Pol II (GSE63965) at the −12 kb region for eRNA transcription, both of which were increased at room temperature (22°C) compared with thermoneutrality (29°C) (Supplemental Fig. S1E; Harms et al. 2015; Emmett et al. 2017). Overall, our eRNA production results further support the presence of an active enhancer at the −12 kb region of the *Ucp1* locus.

To assess whether the −12 kb region is critical for *Ucp1* gene transcription, we performed luciferase reporter assays in differentiated BAT cells by transfecting the luciferase reporter driven by 500 bp of individual peaks (P2–P6) linked to a general minimal promoter (minP) into BAT cells (Fig. 1G, top). As expected, two regions that are known to be critical for *Ucp1* transcription, including P2 at the −2.5 kb enhancer region for CREB/ATF4/PPARγ binding and P4 at the −5.5 kb region for Zc3h10 binding that we previously reported, showed strong luciferase activity compared with empty vector (EV), whereas P3 and P5 did not show any activation (Fig. 1G, left). Importantly, P6 corresponding to the −12 kb region indeed increased the luciferase activity by threefold, compared with P3 and P5. We next transfected P2-, P4-, and P6-linked luciferase constructs into BAT cells, and the cells were differentiated into brown adipocytes and treated with ISO. ISO treatment significantly increased luciferase activity by twofold when driven by P2 or P6 but not by P4, demonstrating the responsiveness of P6 to cAMP–PKA signaling in addition to P2, which is known to be responsive to cAMP–PKA (Fig. 1G, right). We then inserted 1.6 kb of the −12 kb region (from −12,010 to −10,348 bp) into the −5.5 kb *Ucp1* promoter–luciferase construct (Fig. 1H, left) and transfected it into BAT cells, which were then differentiated into brown adipocytes. Indeed, luciferase activity of the chimeric construct transfected cells was increased significantly in both basal and ISO-treated conditions. Next, we tested whether P6 corresponding to the −12 kb region can affect the well-studied −2.5 kb region by generating and using a construct linking P6 with P2. As expected, P6 increased the luciferase activity further by sevenfold compared with P2 only in both basal and stimulated conditions (Fig. 1H, right). These results clearly demonstrate that P6 at the −12 kb region functions as an active enhancer for *Ucp1* transcription and is responsive to cAMP–PKA signaling.

### The −12 kb enhancer is essential for Ucp1 expression in brown adipocytes

**Figure 2.**
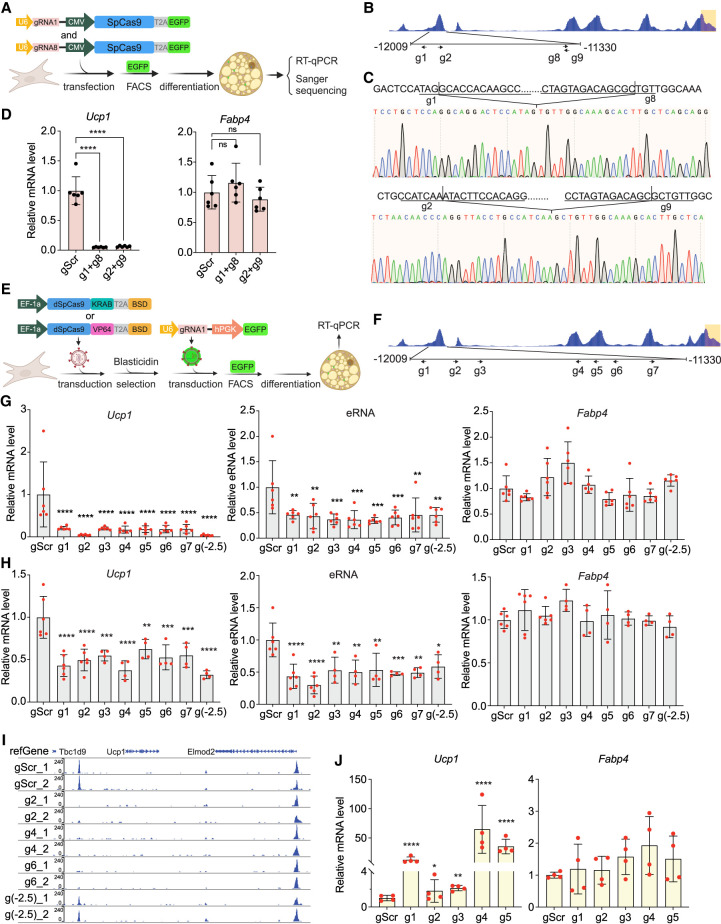
The −12 kb enhancer is required for *Ucp1* gene expression in cultured brown adipocytes. (*A*) Schematic of CRISPR deletion in primary brown adipocytes. (*B*) Two pairs of CRISPR gRNA (g1 and g8 and g2 and g9) were used to delete the −12 kb enhancer. Arrows indicate the location and direction of the gRNA targets. (*C*) Sanger sequencing confirmed the genomic editing by each pair of gRNAs. (*D*) *Ucp1* and *Fabp4* levels in CRISPR-edited primary brown adipocytes (*n* = 8). (*E*) Schematic of CRISPRi and CRISPRa in differentiated BAT cells and primary brown adipocytes. (*F*) Seven gRNAs (g1–g7) targeted on the −12 kb enhancer for CRISPRi. (*G*) *Ucp1* (*left*), eRNA (*middle*), and *Fabp4* (*right*) levels in differentiated BAT CRISPRi cells expressing scramble gRNA (gScr) or gRNA targeting the −12 kb enhancer (g1–g7). gRNA targeting −2.5 kb [g(−2.5)] was used as a positive control (*n* = 6). (*H*) *Ucp1* (*left*), eRNA (*middle*), and *Fabp4* (*right*) levels in primary CRISPRi brown adipocytes expressing gScr or gRNA targeting the −12 kb enhancer (g1–g7). gRNA targeting −2.5 kb [g(−2.5)] was used as a positive control (*n* = 4). (*I*) Genome browser view of chromatin accessibility on the *Ucp1* −12 kb enhancer in primary CRISPRi brown adipocytes by ATAC-seq. (*J*) *Ucp1* (*left*) and *Fabp4* (*right*) levels in differentiated CRISPRa BAT cells expressing gScr or gRNA targeting the −12 kb enhancer (g1–g5) (*n* = 4). (*C*,*E*,*F*,*H*) (*) *P* < 0.05, (**) *P* < 0.01, (***) *P* < 0.005, (****) *P* < 0.001, (ns) nonsignificant based on one-way ANOVA. All error bars represent the mean ± SEM.

Our promoter–reporter assays above demonstrated that the −12 kb region can function as an active enhancer in brown adipocytes in culture. We next sought to test the −12 kb enhancer function in the endogenous genomic context by performing CRISPR deletion in primary brown adipocytes. We transfected pairs of PX459 vectors expressing guide RNAs (gRNAs) flanking the −12 kb enhancer region into primary brown preadipocytes (Cong and Zhang 2015). We then performed FACS to select GFP-positive transfected cells, and these cells were then differentiated into brown adipocytes (Fig. 2A). Locations of each pair of gRNAs (g1 and g8 or g2 and g9) are shown in Figure 2B. Using Sanger sequencing, we confirmed the successful deletion of 527 bp (g1 and g8) or 460 bp (g2 and g9) of the −12 kb enhancer region (Fig. 2C). Compared with the scramble gRNA control (gScr), cells with deletion of the −12 kb enhancer region showed a complete loss of *Ucp1* expression, whereas expression of an adipogenic marker, *Fabp4*, remained the same (Fig. 2D), indicating no change in cell differentiation. These results clearly show that, in the genomic context, the −12 kb region is required for *Ucp1* expression.

To eliminate the possibility of changes in chromatin structure upon deletion of a relatively large DNA segment by CRISPR–Cas9, which makes permanent genomic changes, we next performed CRISPRi by using a dCas9–KRAB (Krüppel-associated box) module (Qi et al. 2013). We generated BAT cells stably expressing dCas9–KRAB (Supplemental Fig. S2A), and the cells were transduced with lentivirus containing gRNA as well as a GFP marker. The gRNA-expressing cells were selected by FACS for GFP and then differentiated into brown adipocytes (Fig. 2E). We used seven gRNAs (g1–g7) targeting the −12 kb region (Fig. 2F), as well as one gRNA targeting a −2.5 kb region [g(−2.5)] as a positive control. Using qPCR, we found that all gRNAs decreased *Ucp1* expression by >80% (Fig. 2G, left), as well as −12 kb eRNA production (Fig. 2G, middle) by >50%, whereas *Fabp4* expression was not significantly changed (Fig. 2G, right). We also performed CRISPRi using primary brown adipocytes by isolating SVF from BAT of dCas9–KRAB transgenic mice (Jackson strain 030000) (Yoneshiro et al. 2019). Cells were transduced with lentivirus containing gRNA and GFP and sorted for GFP-positive cells. These cells were then differentiated into brown adipocytes. Using qPCR, we observed a significant decrease in both *Ucp1* mRNA and eRNA levels but not *Fabp4* mRNA levels (Fig. 2H). We then performed ATAC-seq in these CRISPRi primary brown adipocytes and found that cells transduced with gRNA targeting the −12 kb enhancer region eliminated the −12 kb ATAC peak (Fig. 2I). These results indicate that the −12 kb enhancer is essential for *Ucp1* expression in the genome context, and repressing this enhancer leads to a more condensed chromatin state and limits DNA accessibility in this region. Interestingly, we found that gRNA targeting the −2.5 kb region could also reduce the −12 kb ATAC peak by 60% (Fig. 2I), indicating a physical proximity in 3D space between the −12 and −2.5 kb regions, supporting the existence of a loop between −12 kb enhancer region and the promoter region. Worth mentioning is that the ATAC-seq peaks in cultured brown adipocyte were quite different from those we detected in brown adipose tissue. The −12 kb peak in differentiated primary brown adipocytes was much stronger than other peaks on the *Ucp1* genome, similar to those reported by other researchers in primary brown adipocytes (Jang et al. 2021).

The results from CRISPRi on the −12 kb enhancer showed a clear silencing of *Ucp1*, and we next used CRISPRa to further examine the function of the −12 kb enhancer. We generated BAT cells stably expressing dCas9–VP64, a transcriptional activator containing four tandem copies of VP16 (Supplemental Fig. S2B). These cells were transduced individually with lentivirus containing five different gRNAs targeting the −12 kb enhancer and differentiated into brown adipocytes (Fig. 2E). The *Ucp1* expression levels in these cells wwere assessed by qPCR. Three out of the five gRNAs showed an ∼10-fold to 50-fold increase in *Ucp1* expression compared with the gScr control (Fig. 2J). Overall, we conclude that the −12 kb region is an active enhancer that is indispensable for *Ucp1* expression.

### The −12 kb enhancer is critical for thermogenesis in vivo

**Figure 3.**
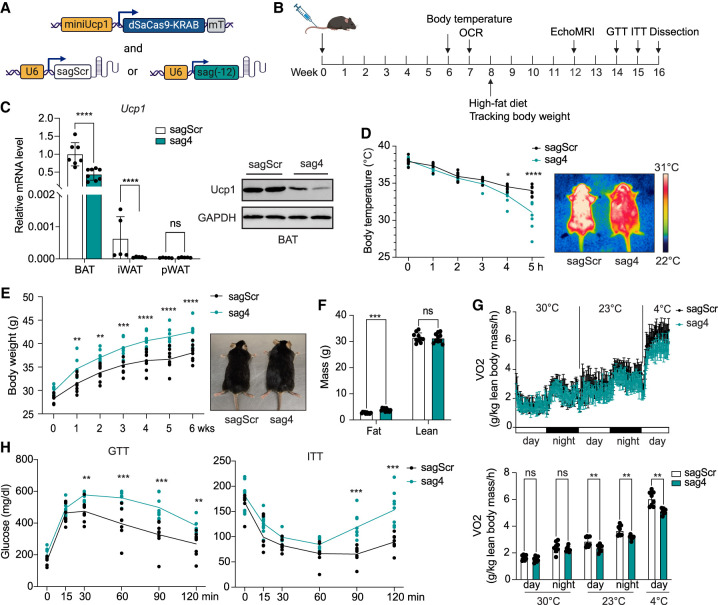
Repression of the −12 kb enhancer suppresses thermogenesis in mice. (*A*) Diagram of CRISPRi construction. (*B*) Schematic protocol for characterizing the metabolic phenotypes of mice. (OCR) Oxygen consumption rates, (GTT) glucose tolerance test, (ITT) insulin tolerance test. (*C*) *Ucp1* RNA levels in BAT (*n* = 8), iWAT (*n* = 5), and pWAT (*n* = 5) (*left*) and protein levels in BAT (*right*) of gScr and g(−12) CRISPRi mice. (*D*) Rectal temperature changes during 5 h of cold exposure (*left*) and infrared thermograph (*right*) of gScr and g(−12) CRISPRi mice (*n* = 8). (*E*) Body weight changes during 8 weeks of high-fat diet feeding (*left*) and a picture of gScr and g(−12) CRISPRi mice (*right*) (*n* = 8) are shown. (*F*) Body composition of gScr (*n* = 10) and g(−12) (*n* = 8) CRISPRi mice. (*G*, *top*) VO_2_ measured by CLAMS at serial time point during thermoneutrality (30°C), ambient temperature (23°C), and cold challenge (4°C). (*Bottom*) Averaged VO_2_ as measured by CLAMS (*n* = 8). (*H*) GTT and ITT in gScr (*n* = 7) and g(−12) (*n* = 9) CRISPRi mice. (*C*–*H*) (*) *P* < 0.05, (**) *P* < 0.01, (***) *P* < 0.005, (****) *P* < 0.001, (ns) nonsignificant based on two-way ANOVA. All error bars represent the mean ± SEM. (

To assess the physiological relevance of our initial findings in cultured brown adipocytes, we conducted CRISPRi by adeno-associated virus (AAV)-mediated systemic delivery of dSaCas9–KRAB and gRNAs targeting the −12 kb enhancer in mice. Due to the size limitation of AAV8, we used *Staphylococcus aureus* Cas9 (3156 bp; designated as SaCas9) to distinguish it from *Streptococcus pyogenes* Cas9 (SpCas9), which we used above in cultured cells. dSaCas9–KRAB was driven by the *Ucp1* promoter to limit expression to thermogenic adipocytes (Kozak et al. 1994; Jimenez et al. 2013). To further ensure elimination of hepatic expression, we inserted four tandem repeats of the miR-122a target sequence (mT) downstream from the stop codon (Fig. 3A; Kozak et al. 1994). We first tested various SaCas9-compatible gRNAs (sags) designed to target the −12 kb enhancer (Supplemental Fig. S3A) by transducing gRNA lentivirus into BAT cells stably expressing dSaCas9–KRAB. Two gRNAs targeting the −2.5 kb region, which reduced *Ucp1* mRNA levels by ∼50%, and scramble gRNA (sagScr) were used as positive and negative controls, respectively (Supplemental Fig. S3B). Among all gRNAs that were effective in reducing *Ucp1* mRNA levels, we selected sag4 (targeting at −11,522 bp) for its consistent and significant reduction of *Ucp1* mRNA levels by >60% without affecting *Fabp4* expression (Supplemental Fig. S3B). We thus administered mice with AAV8 containing *Ucp1* promoter-driven dSaCas9–KRAB along with sag4 via tail vein injection. Mice were examined at the indicated time points (Fig. 3B). Reduction in *Ucp1* mRNA levels was observed in both BAT and iWAT by qPCR (Fig. 3C, left), and the reduction of Ucp1 protein in BAT was confirmed by immunoblotting (Fig. 3C, right). Mice injected with dSaCas9–KRAB and sag4 were unable to maintain core body temperature after 4 h of cold exposure (Fig. 3D, left) compared with sagScr mice. Infrared imaging of sag4-injected mice also showed significantly lower body temperature (Fig. 3D, right). Furthermore, these CRISPRi mice exhibited higher body weights when on a high-fat diet for 6 weeks (Fig. 3E), with increased adipose tissue mass as measured by EchoMRI (Fig. 3F), without significant changes in food intake (Supplemental Fig. S3C). Moreover, these mice showed lower oxygen consumption rates (OCRs) by CLAMS during both the day and night when maintained at 23°C, and the differences in OCR were even greater at 4°C (Fig. 3G, top and bottom), demonstrating a defective thermogenesis upon blocking of −12 kb enhancer activity in mice. These sag4 CRISPRi mice also showed impaired glucose tolerance and reduced insulin sensitivity compared with control mice (Fig. 3H).

**Figure 4.**
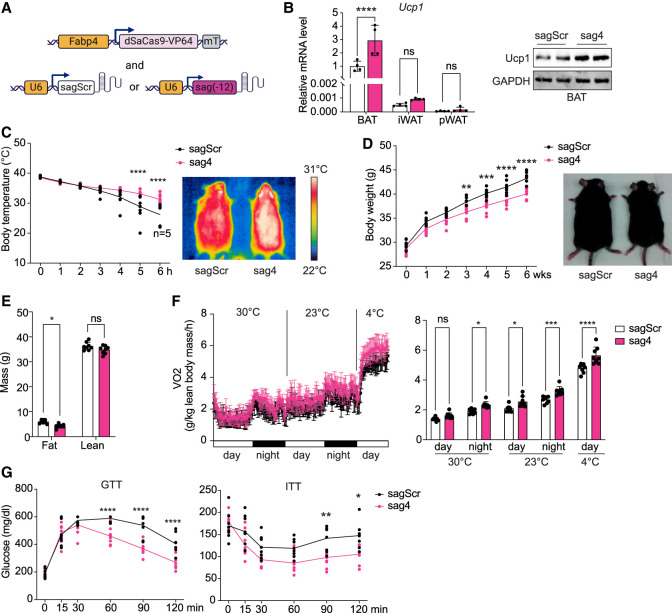
Activation of the Ucp1 −12 kb enhancer by CRISPRa promotes thermogenesis. (*A*) Diagram of CRISPRa construction. (*B*) Ucp1 RNA levels in BAT, iWAT, and pWAT (*left*) and protein levels in BAT (*right*) of gScr and g(−12) CRISPRa mice (*n* = 4). (*C*) Rectal temperature changes during 6 h of cold exposure (*left*) and infrared thermograph (*right*) of gScr (*n* = 5) and g(−12) (*n* = 8) CRISPRa mice. (*D*) Body weight changes during 6 weeks of high-fat diet feeding (*left*) and a picture of gScr and g(−12) CRISPRa mice (*right*) (*n* = 8) are shown. (*E*) Body composition of gScr and g(−12) CRISPRa mice (*n* = 8). (*F*, *left*) VO_2_ as measured by CLAMS at serial time points during thermoneutrality (30°C), ambient temperature (23°C), and cold challenge (4°C). (*Right*) Averaged VO_2_ as measured by CLAMS (*n* = 8). (*G*) GTT and ITT in gScr and g(−12) CRISPRa mice (*n* = 8). (*B*–*G*) (*) *P* < 0.05, (**) *P* < 0.01, (***) *P* < 0.005, (****) *P* < 0.001, (ns) nonsignificant based on two-way ANOVA. All error bars represent the mean ± SEM.

Next, we conducted CRISPRa experiments in mice using an AAV containing a minimal Fabp4 promoter-driven dSaCas9–VP64 along with an AAV containing gRNA (Fig. 4A). Due to the size limitations of the AAV vector, the adiponectin promoter could not be used together with dCas9–VP64; therefore, we used the minimal *Fabp4* promoter. Although *Fabp4* expression has been reported in macrophages (Furuhashi and Hotamisligil 2008), intravascular delivery of AAV8 did not lead to transduction into macrophages in eWAT, iBAT, the liver, or peripheral blood (Jimenez et al. 2013). We tested five gRNAs targeting the −12 kb enhancer in cultured cells by transducing the gRNA-expressing lentivirus to a BAT cell line stably expressing dSaCas9–VP64 to identify the most effective gRNA for activation, and sag4 was selected for in vivo experiments for its consistent and efficient activation of *Ucp1* more than sixfold (Supplemental Fig. S4). An increase in *Ucp1* mRNA and protein levels was observed in both BAT and iWAT (Fig. 4B). Mice injected with dSaCas9–VP64 and sag4 exhibited higher core body temperatures after 4 h of cold exposure compared with mice injected with the sagScr control (Fig. 4C, left). Infrared imaging confirmed that sag4-injected mice maintained significantly higher body temperatures (Fig. 4C, right). Additionally, these CRISPRa mice showed lower body weights on a high-fat diet for 6 weeks (Fig. 4D) and a reduction in adipose tissue mass, as measured by EchoMRI (Fig. 4E). Furthermore, oxygen consumption rates (OCRs) measured by CLAMS were higher in these mice during both the day and night at 23°C, with even greater differences observed at 4°C (Fig. 4F). Finally, sag4 CRISPRa mice displayed higher tolerance to glucose and higher insulin sensitivity compared with control mice (Fig. 4G). Altogether, we conclude that the −12 kb enhancer plays a critical role in regulating *Ucp1* expression. Activation of this enhancer in adipose tissue significantly influences metabolic outcomes, including body temperature maintenance, fat mass reduction, and improved glucose and insulin homeostasis.

### Etv4 directly binds to the −12 kb enhancer to activate the *Ucp1* gene

**Figure 5.**
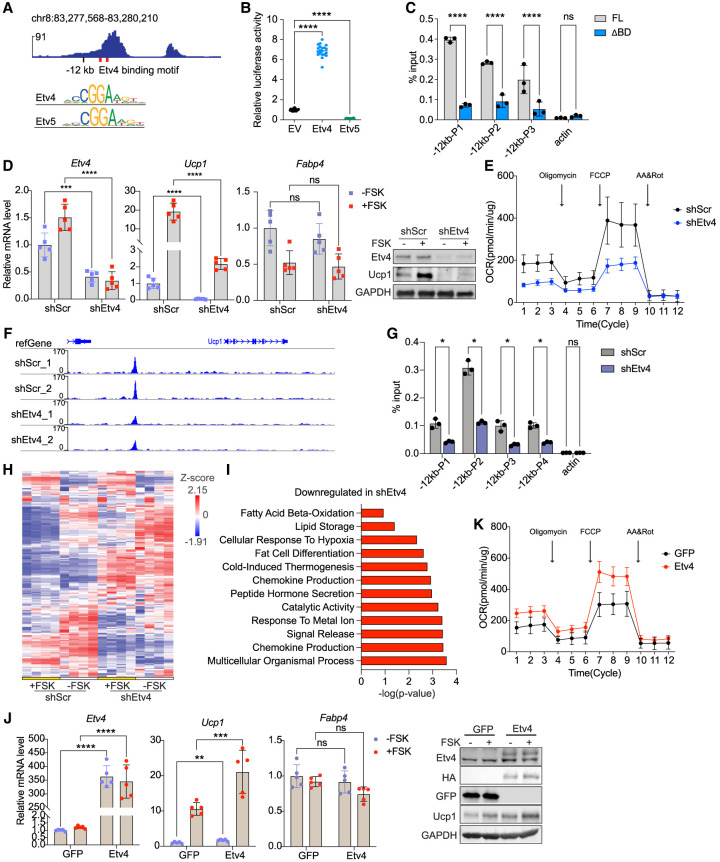
Etv4 activates Ucp1 by binding at the −12 kb enhancer and promotes chromatin accessibility. (*A*) Two Etv4 binding motifs on the Ucp1 −12 kb enhancer (*top*) and sequence logo of the Etv4 and Etv5 binding motif (*bottom*). (*B*) Luciferase activity is driven by the −12 kb enhancer when cotransfected with *Etv4* or *Etv5* in 293T cells (*n* = 20). (*C*) ChIP-qPCR of full-length (FL) and DNA-binding domain-deleted (ΔBD) Etv4 binding on the Ucp1 −12 kb enhancer in brown adipocytes. (*D*, *left*) *Etv4*, *Ucp1*, and *Fabp4* RNA levels in scramble and *Etv4* knockdown brown adipocytes with or without forskolin (FSK) treatment (*n* = 5). (*Right*) Protein levels of the indicated genes in brown adipocytes. (*E*) OCR of scramble and *Etv4* knockdown brown adipocytes (*n* = 5). (*F*) Genome browser results of ATAC-seq on the Ucp1 genome locus in scramble or *Etv4* knockdown brown adipocytes. (*G*) H3k27ac ChIP-qPCR using four primer pairs targeting the −12 kb enhancer in scramble or *Etv4* knockdown brown adipocytes. Primer targeting actin was used as a negative control. (*H*) Heat map showing the changes in gene expression profiles in scramble and *Etv4* knockdown brown adipocyte with or without FSK treatment. (*I*) Gene ontology analysis of the downregulated genes in *Etv4* knockdown brown adipocytes. (*J*, *left*) *Etv4*, *Ucp1*, and *Fabp4* levels in GFP control or Etv4-overexpressing brown adipocytes with or without FSK treatment (*n* = 5). (*Right*) Protein levels of the indicated genes in brown adipocytes. (*K*) OCR of GFP control or Etv4-overexpressing brown adipocytes (*n* = 5). (*) *P* < 0.05, (**) *P* < 0.01, (***) *P* < 0.005, (****) *P* < 0.001, (ns) nonsignificant based on one-way ANOVA (*B*) or two-way ANOVA (*C*,*D*,*G*,*J*). All error bars represent the mean ± SEM.

Because enhancer function is determined by binding of specific transcription factors, we sought to identify and characterize those factors that bind to the −12 kb region and enable it to function as an enhancer for *Ucp1* transcriptional activation. We began by examining various publicly available ChIP-seq data sets in an attempt to find potential transcription factors that may bind to the −12 kb enhancer region. Through a peak set overlap analysis of publicly available ChIP-seq data (Mei et al. 2017; Zheng et al. 2019), we found several factors that can bind to the −12 kb enhancer region of *Ucp1* gene, including chromatin modifiers and transcription factors, such as Etv5 (Supplemental Fig. S5A). We chose to focus on ETV family transcription factors because we detected two Etv4 binding sites within the −12 kb enhancer region (Fig. 5A, top). Given that Etv4 and Etv5 share high similarity in their binding sites (Fig. 5A, bottom), we cotransfected the −12 kb enhancer-driven luciferase reporter construct with either *Etv4* or *Etv5* expression vector into 293FT cells for luciferase assay. Cotransfection of *Etv4* resulted in a sevenfold increase in luciferase activity compared with the empty vector (EV) control, whereas *Etv5* did not increase luciferase activity (Fig. 5B). These findings suggest that Etv4, but not Etv5, functions as an activator at the −12 kb *Ucp1* enhancer region. Next, we performed ChIP-qPCR for Etv4 in brown adipocytes to test whether Etv4 binds endogenously to the −12 kb enhancer region. We generated two BAT cell lines stably expressing either full-length or DNA-binding domain (amino acids 342–384)-deleted (ΔBD) Etv4 (Supplemental Fig. S5B). ChIP-qPCR using three different primer pairs targeting the −12 kb enhancer region revealed a strong binding of the full-length Etv4, but not ΔBD Etv4, at this region. Primers targeting GAPDH as a negative control did not show any Etv4 binding (Fig. 5C). These results support the notion that Etv4 directly binds to the *Ucp1* −12 kb enhancer through its C-terminal DNA binding domain to activate the Ucp1 gene.

### Etv4 promotes thermogenesis in brown adipocytes in vitro

Because Etv4 can activate *Ucp1* gene expression through directly binding to the −12 kb *Ucp1* enhancer, we next examined the impact of *Etv4* on thermogenesis in brown adipocytes. We first performed *Etv4* knockdown by transducing brown adipocytes with lentivirus expressing either shRNA targeting *Etv4* (shEtv4) or a scrambled control shRNA (shScr). The shEtv4 lentivirus efficiently reduced *Etv4* RNA levels by 60% (Fig. 5D, left), with nearly undetectable Etv4 protein (Fig. 5D, right). Knockdown of *Etv4* led to an eightfold decrease in *Ucp1* mRNA levels under both basal and forskolin (FSK)-stimulated conditions (Fig. 5D, middle), rendering Ucp1 protein nearly undetectable (Fig. 5D, right), whereas *Fabp4* expression remained unchanged (Fig. 5D, middle). Seahorse experiments on *Etv4* knockdown brown adipocytes revealed significantly reduced OCR, indicating impaired thermogenic activity (Fig. 5E). To further explore the role of Etv4 in enhancer function, we performed ATAC-seq on shEtv4 brown adipocytes. We detected an ∼60% reduction in chromatin accessibility at the −12 kb enhancer in shEtv4 cells compared with shScr control cells, suggesting involvement of Etv4 in chromatin opening and enhancer activation at this locus (Fig. 5F). Consistently, ChIP-qPCR with these cells revealed a significant decrease in H3K27ac accumulation at the −12 kb enhancer upon *Etv4* knockdown (Fig. 5G), supporting the role of Etv4 in histone modification and enhancer activation at this region. Moreover, to assess the genome-wide changes in gene expression, we performed RNA-seq in Etv4 knockdown adipocytes. As expected, *Etv4* depletion drastically altered the gene expression profile (Fig. 5H). GO term analysis revealed significant downregulation of genes associated with fatty acid oxidation, lipid storage, and thermogenesis (Fig. 5I).

Next, we conducted a gain-of-function study by overexpressing *Etv4* in brown adipocytes. Differentiated BAT cells were transduced with lentivirus expressing HA-tagged GFP or Etv4. *Etv4* overexpression was confirmed by RT-qPCR (Fig. 5J, left). Ectopic expression of HA-tagged GFP and Etv4 was clearly detected by immunoblotting (Fig. 5J, right). Indeed, both Ucp1 mRNA and protein levels were elevated in these Etv4-overexpressing cells compared with GFP control cells, and this increase was observed under both basal and FSK-stimulated conditions (Fig. 5J). Moreover, OCR measured by Seahorse assay in these Etv4-overexpressing cells showed a higher OCR, reflecting enhanced Ucp1 function for thermogenesis (Fig. 5K). Altogether, these results show that Etv4 promotes Ucp1 expression by binding to the −12 kb region and that Etv4 is essential for −12 kb enhancer function and Ucp1 transcription and thus thermogenesis in brown adipocytes in culture.

### Etv4 promotes thermogenesis and energy expenditure in mice

**Figure 6.**
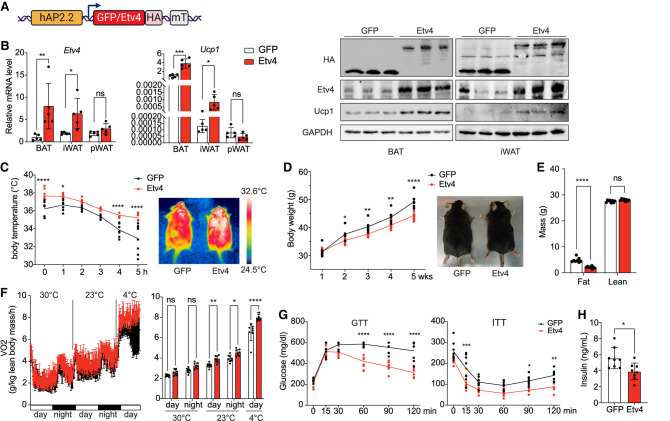
Overexpression of Etv4 in mouse adipose tissue promotes thermogenesis. (*A*) Diagram of AAV-mediated expression of GFP or Etv4 under a human adiponectin minimal promoter with a miR-122 target sequence (mT). (*B*) *Etv4* and *Ucp1* RNA levels in the indicated tissues in GFP control or Etv4-overexpressing mice (*left*) (*n* = 5) and protein levels in BAT and iWAT (*right*). (*C*) Rectal temperature as measured during 5 h of cold exposure (*left*) (*n* = 8) and infrared thermograph of GFP control and Etv4-overexpressing mice (*right*). (*D*) Body weight changes during 8 weeks of high-fat diet feeding (*left*) (*n* = 8) and a picture of GFP control and Etv4-overexpressing mice (*right*) are shown. (*E*) Body composition of GFP control and Etv4-overexpressing mice (*n* = 8). (*F*) VO_2_ as measured by CLAMS at serial time points during thermoneutrality (30°C), ambient temperature (23°C), and cold challenge (4°C) (*left*) and averaged VO_2_ as measured by CLAMS (*right*) (*n* = 8). (*G*) GTT and ITT in GFP control and Etv4-overexpressing mice (*n* = 8). (*H*) Fasting insulin levels in GFP control and Etv4-overexpressing mice (*n* = 8). (*B*–*H*) (*) *P* < 0.05, (**) *P* < 0.01, (***) *P* < 0.005, (****) *P* < 0.001, (ns) nonsignificant based on two-way ANOVA. All error bars represent the mean ± SEM.

To examine the physiological effect of Etv4, we conducted an in vivo gain-of-function study by systemically delivering AAV8 carrying HA-tagged Etv4 or GFP control driven by an adiponectin promoter/enhancer to ensure restricted expression in mature adipocytes (Fig. 6A; Segawa et al. 2009; Chella Krishnan et al. 2021). These mice were then examined for the impact of Etv4 on thermogenesis in BAT, as well as browning of subcutaneous WAT. *Etv4* mRNA levels were elevated approximately eightfold in BAT and threefold in iWAT, whereas no significant changes were observed in perigonadal WAT (pWAT). Correspondingly, Etv4 protein levels were markedly increased in BAT and iWAT. Importantly, *Ucp1* mRNA expression exhibited a fourfold increase in BAT and a fivefold increase in iWAT in Etv4-overexpressing mice (Fig. 6B, left). Consistent with these findings, Ucp1 protein levels were also significantly elevated in Etv4-overexpressing mice (Fig. 6B, right).

We then examined the metabolic effects of Etv4 in mice. Etv4-overexpressing mice exhibited greater cold tolerance and were able to maintain a higher core body temperature compared with control mice upon cold exposure (Fig. 6C, left). Infrared imaging confirmed a significantly higher body temperature in Etv4-overexpressing mice (Fig. 6C, right). Moreover, Etv4 mice on a high-fat diet exhibited significantly lower body weights (Fig. 6D), accompanied by decreased fat mass, as measured by EchoMRI (Fig. 6E). These mice also showed increased OCR as measured by CLAMS, especially upon cold exposure (Fig. 6F). Additionally, Etv4 mice showed enhanced glucose tolerance and improved insulin sensitivity (Fig. 6G), with lower serum insulin levels as measured after 6 h of fasting (Fig. 6H). In conclusion, Etv4 promotes thermogenesis and enhances systemic metabolism by upregulating *Ucp1* expression, thereby providing protection against diet-induced obesity and insulin resistance.

**Figure 7.**
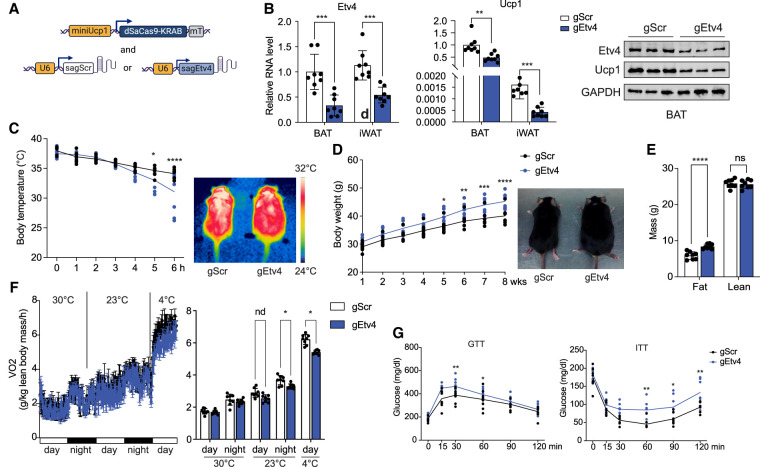
Repression of Etv4 inhibits thermogenesis in mice. (*A*) Diagram of AAV-mediated expression of dcas9–KRAB under minimal Ucp1 promoter with a miR-122 target sequence (mT) and of scramble and *Etv4* gRNA expression under a U6 promoter. (*B*) *Etv4* and *Ucp1* RNA levels (*left*) (*n* = 8) and protein levels (*right*) in the indicated tissues of scramble and *Etv4* CRISPRi mice. (*C*) Rectal temperature as measured during 6 h of cold challenge (*left*) (*n* = 8) and an infrared thermograph of scramble and *Etv4* CRISPRi mice (*right*) are shown. (*D*) Body weight changes during 8 weeks of high-fat diet feeding (*left*) (*n* = 8) and a picture of scramble and *Etv4* CRISPRi mice (*right*) are shown. (*E*) Body composition of scramble and *Etv4* CRISPRi mice (*n* = 8). (*F*) VO_2_ as measured by CLAMS at serial time points during thermoneutrality (30°C), ambient temperature (23°C), and cold challenge (4°C) (*left*) and averaged VO_2_ as measured by CLAMS (*right*) (*n* = 8). (*G*) GTT and ITT in scramble (*n* = 7) and *Etv4* (*n* = 8) CRISPRi mice. (*B*–*G*) (*) *P* < 0.05, (**) *P* < 0.01, (***) *P* < 0.005, (****) *P* < 0.001, (ns) nonsignificant based on two-way ANOVA. All error bars represent the mean ± SEM.

We next performed a loss-of-function study in mice using CRISPRi to repress Etv4 expression in Ucp1^+^ cells (Fig. 7A, left). First, 12 gRNAs targeting the *Etv4* promoter region with opened chromatin as shown in our ATAC-seq data were tested in BAT cells for their efficiency in suppressing *Etv4* expression (Supplemental Fig. S6A). High repression efficiency was detected with gEtv4-3, gEtv4-4, and gEtv4-5 (Supplemental Fig. S6B). We then intravenously injected mice with AAVs carrying a pooled gEtv4-3, gEtv4-4, and gEtv4-5 (gEtv4) or a scramble control (gScr), along with an AAV containing dSaCas9–KRAB. *Etv4* CRISPRi mice exhibited significantly reduced *Etv4* expression (70% in BAT and 40% in iWAT) at RNA levels. Correspondingly, Etv4 protein levels in BAT were significantly reduced as shown by immunoblotting. More importantly, Ucp1 expression in BAT was also markedly reduced in *Etv4* CRISPRi mice (Fig. 7B). *Etv4* CRISPRi mice also displayed impaired cold tolerance, as indicated by a significantly lower core body temperature after 5 h of cold exposure. Infrared imaging similarly showed a lower body temperature of *Etv4* CRISPRi mice (Fig. 7C). Furthermore, body weights of *Etv4* CRISPRi mice were significantly higher than those of gScr control mice after 5 weeks on a high-fat diet (Fig. 7D). EchoMRI analysis revealed an increase in fat mass in *Etv4* CRISPRi mice (Fig. 7E). Metabolic analysis demonstrated that *Etv4* CRISPRi mice had significantly reduced OCR as measured by CLAMS at night at 23°C, with further declines observed during cold challenge (Fig. 7F). Additionally, *Etv4* CRISPRi mice exhibited impaired glucose tolerance and reduced insulin sensitivity (Fig. 7G). Collectively, these findings from gain- and loss-of-function studies demonstrate that Etv4 binding to the −12 kb enhancer is critical for maintaining high-level Ucp1 expression in BAT and for promoting browning of subcutaneous WAT. Overall, Etv4 binding to the −12 kb enhancer is essential for sustaining body temperature and increasing energy expenditure, thereby preventing diet-induced obesity and insulin resistance.

## Discussion

Thermogenic adipose tissues (brown or beige fat), with their remarkable ability for increasing energy expenditure and insulin sensitivity, have emerged as promising targets for therapeutic interventions against obesity and related metabolic diseases. Gaining a deeper understanding of the molecular mechanisms regulating BAT thermogenesis and metabolism is of critical importance. Ucp1 serves as a key biological and functional marker of both brown and beige adipocytes. With its capacity to uncouple oxidative phosphorylation from ATP synthesis, Ucp1 can increase thermogenesis and energy dissipation, highlighting its potential as a therapeutic target. Although most studies on *Ucp1* transcription focus on the −5 kb upstream region, including the −2.5 kb region and its associated transcription factors or coregulators, our study identified another *Ucp1* enhancer located −12 kb upstream of the *Ucp1* gene. We demonstrated that this enhancer is essential for *Ucp1* transcription through chromatin looping. Furthermore, we showed that Etv4 binds to this enhancer, facilitating chromatin opening and promoting transcriptional activation of the *Ucp1* gene.

Our ATAC-seq analysis of BAT revealed multiple chromatin openings at the *Ucp1* genomic locus, indicating a complex regulation of *Ucp1* transcription. Notably, these chromatin openings were absent in WAT. The chromatin accessibility at the Ucp1 locus remained stable in BAT under both thermoneutrality and after 2 days of cold exposure (data not shown). The Lazar laboratory (Inoue et al. 2024) has reported that cold exposure can induce chromatin opening at specific enhancer regions but not at the *Ucp1* genomic locus. Even in BAT from mice subjected to 5 months of high-fat diet feeding, no detectable changes in chromatin status were observed at the *Ucp1* promoter and enhancer regions.

High enrichment of H3K27ac and H3K4me1 at this −12 kb enhancer region indicates its role as an active enhancer. Although some studies suggested the presence of an open chromatin at this region, none have investigated its function as an enhancer (Emmett et al. 2017; Lai et al. 2017; Rajbhandari et al. 2018). Our current study demonstrated that the −12 kb enhancer forms a loop with the proximal promoter region of the *Ucp1* gene. Functional assays using promoter–reporter constructs demonstrated that the −12 kb region exhibits strong transcriptional activation potential, comparable with the well-established function of the −2.5 kb region. Moreover, deletion or inhibition of this −12 kb enhancer using CRISPR or CRISPRi in mice significantly repressed *Ucp1* transcription, underscoring its essential role in regulating *Ucp1* expression in vivo. Although previous studies reported that the 221 bp enhancer region between −2530 and −2310 bp is sufficient to drive *Ucp1* gene expression specifically in brown adipocytes (Cassard-Doulcier et al. 1998), our findings suggest that the −2.5 kb region likely contributes more to tissue specificity than transcriptional strength, whereas the −12 kb region appears to have a greater influence on transcriptional strength than tissue specificity, based on our results from CRISPR and CRISPRi on the −12 kb enhancer. This observation aligns with earlier reports that transcriptional strength often correlated with enhancer activity (Henriques et al. 2018; Mikhaylichenko et al. 2018; Field and Adelman 2020). Importantly, our results reveal that the −12 kb enhancer collaborates with the −2.5 kb region in vivo to achieve the full transcriptional capacity of the *Ucp1* gene. This is evidenced by a 50-fold increase in *Ucp1* expression upon CRISPRa of the −12 kb enhancer and a 90% reduction in expression following CRISPRi suppression. We also explored the presence of a long-distance enhancer for *UCP1* in the human genome. There is a distal enhancer-like signature (enhD) shown by UCSC at −15,747 bp (Supplemental Fig. S7A). Interestingly, an ETV4 motif was also found in the enhD region at −15,436 bp. We also validated its function by CRISPRa using five gRNAs targeting the 340 bp enhD region in human beige adipocytes. All gRNA transduced cells exhibited elevated *UCP1* expression, with no significant change in *FABP4* expression (Supplemental Fig. S7B). These results indicate that the −12 kb enhancer in mice and its human counterpart play critical roles in regulating *Ucp1* expression. This highlights the potential of targeting this enhancer as a therapeutic strategy for addressing metabolic disorders, such as obesity and insulin resistance.

We initially analyzed publicly available ChIP-seq data to study the regulation through the −12 kb enhancer and revealed several factors that bind to the −12 kb *Ucp1* enhancer. These include EP300 (P300), JunD, PPARγ, MED1, KDM3A, TEAD1 (TEF-1/TCF-13), POLR2A (RNA polymerase II large subunit), and SMARCB1 (a SWI/SNF subunit) (Supplemental Fig. S5A). Our focus was on transcription factors that could specifically regulate brown adipocyte function through directly binding to the −12 kb *Ucp1* enhancer. Factors such as EP300, MED1, KDM3A, and SMARCB1 were excluded from consideration because they do not bind DNA directly. Initially, we tested JunD, but our preliminary analyses did not yield positive results (data not shown). We then focused on Etv4, a member of the ETS family of transcription factors, based on its potential role in metabolic regulation. Notably, phenome-wide association studies (PheWAS) of *Etv4* revealed a significant number of genomic variants associated with metabolic traits (*P*-value < 0.01), suggesting its involvement in metabolic diseases (Supplemental Fig. S7C).

The ETS family of transcription factors plays critical roles in various biological processes, including development, proliferation, cell differentiation, and survival. Interestingly, Etv4 has been previously reported to induce PPARγ expression during adipocyte differentiation (Park et al. 2010). In this study, we uncovered a novel function of Etv4 in thermogenesis. Specifically, we demonstrated that Etv4 binds to the −12 kb enhancer of the *Ucp1* gene, which is required for the histone modification and chromatin opening of this region, indicating a role of Etv4 in chromatin remodeling in brown adipose tissue. ETS family members have been reported to recruit histone acetylase or deacetylase at the enhancer region to alter chromatin structure for activation or repression, respectively (Koyano-Nakagawa et al. 2022). Previous studies of human prostate cancers have shown that ETS family transcription factors interact with the SWI/SNF(BAF) ATP-dependent chromatin remodeling complex to redirect BAF complex targeting (Sandoval et al. 2018; Pham et al. 2019). However, although we found an impact of Etv4 on H3K27ac deposition and chromatin accessibility in brown adipocytes, whether it depends on interaction with the BAF complex remains unclear. Future studies using in situ capture and mass spectrometry may help address this question.

Other transcription factors, such as PPARγ, Ebf2, and Cebpβ, which are known to regulate adipogenesis and thermogenesis, were reported to bind at the −12 kb enhancer region (MacDougald et al. 1995; Shapira et al. 2017; Shen et al. 2020; Inoue et al. 2024). Therefore, we performed coimmunoprecipitation (co-IP) experiments in BAT injected with AAV carrying HA-tagged Etv4 driven by an adiponectin promoter. The co-IP analyses revealed a clear interaction between Etv4 and PPARγ or Ebf2 but not with Cebpβ (Supplemental Fig. S7D). The lack of interaction with Cebpβ could possibly be due to the fact that Cebpβ binds the *Ucp1* genome locus only upon cold exposure, as reported by Inoue et al. (2024). Regardless, potential cooperation of Etv4 with these transcription factors involved in protein–protein interaction at the −12 kb enhancer needs further investigation.

## Materials and methods

### Mouse models

C57BL/6 J mice and dCas9-KRAB transgenic mice were housed in a 12:12 light–dark cycle with food and water provided ad libitum. Unless stated otherwise, mice used for AAV injection were male C57BL/6 J at 8 weeks of age. Mice were fed a chow diet or a palatable high-fat diet (45% fat-derived calories; Dyets) ad libitum. All animal studies were carried out in accordance with the University of California, Berkeley, Animal Care and Use Committee and Office of Laboratory Animal Care regulations.

### Cell culture

HEK293, AAVPro293T, and 3T3-L1 cells were maintained in DMEM with 10% FBS and 1% penicillin–streptomycin (P/S). The immortalized murine brown preadipocyte line (BAT cells) was a generous gift from Shingo Kajimura (Galmozzi et al. 2014). Cells were maintained in DMEM supplemented with 10% FBS and 1% P/S. Brown adipocyte differentiation was performed as described by Kajimura et al. (2008). Specifically, confluent preadipocytes were differentiated by adding differentiation cocktails (5 µg/mL insulin, 1 nM T3, 125 µM indomethacin, 0.5 mM 3-isobutyl-1-methylxanthine, 5 µM dexamethasone, 0.5 µM rosiglitazone) for 2 days. Cells were then switched to maintenance medium containing 10% FBS, P/S, insulin, T3, and rosiglitazone. To induce thermogenic genes, cells were differentiated for 5 days and then stimulated with 10 mM isoproterenol or 10 mM forskolin for 4 h for RNA or for 6 h for protein. 3T3-L1 cells were differentiated after confluency by adding 10 µg/mL insulin, 0.5 mM 3-isobutyl-1-methylxanthine, and 5 µM dexamethasone for 2 days, and then cells were maintained in DMEM with 10 µg/mL insulin. Human primary preadipocytes were purchased from ZenBio (SP-F-SL) and cultured in basic medium (DMEM/F12 with 10% FBS, 10 mM HEPES, 1% P/S) supplemented with 5 ng/mL bFGF and 5 ng/mL rhEGF. Beige adipogenesis was induced 2 days after confluency with basic medium in the presence of the aforementioned differentiation cocktail for 3 days (days 0–3). Cells were then switched to basic medium with insulin, T3, and rosiglitazone for 5 days. 293FT cells were used for lentivirus packaging. AAVpro293T cells (Takara) were used for AAV packaging.

### Primary brown preadipocyte isolation

Brown adipose tissues of dcas9-KRAB mice were minced and digested with collagenase type II (Gibco 17101015) in HBSS buffer with 3% BSA for 45 min at 37°C with shaking. The cell suspension was then passed through a 100 mm cell strainer and spun at 500*g* for 5 min. The cell pellet was resuspended in HBSS buffer and passed through 70 and 40 mm cell strainers. All the procedures were performed in a cell culture hood to avoid contamination. Cells were resuspended in growth medium and seeded in a 10 cm cell culture plate for overnight growing. The next day, the cell culture plate was washed three times with PBS to remove the floating red blood cells and immune cells. The cells were maintained in regular medium and subjected to transduction.

### Chromosome conformation capture and qPCR

Chromosome conformation capture (3C) experiments were performed according to the published protocol with some optimization (Hagège et al. 2007). Briefly, 5 × 10^7^ differentiated BAT cells were cross-linked with 1% formaldehyde for 10 min at room temperature and quenched in 0.125 M glycine for 5 min. Nuclei were extracted in lysis buffer (10 mM Tris-HCl at pH 7.5, 10 mM NaCl, 5 mM MgCl_2_, 0.1 mM EGTA, proteinase inhibitor) for 10 min on ice and then pelleted. For 3C experiments in brown adipocytes from tissue, brown adipose tissues were isolated by digesting tissue in collagenase type II (Gibco 17101015) in HBSS buffer for 1 h and gently removing all the fibroblasts by spinning at 500*g* for 5 min. The floating adipocytes were transferred to new tubes and lysed in 1/10 vol of 10× cell lysis buffer (100 mM Tris-HCl at pH 7.5, 100 mM NaCl, 50 mM MgCl_2_, 1 mM EGTA, proteinase inhibitor) for 10 min on ice and then pelleted. BstYI digestion was carried out at overnight 37°C. T4 ligase was added to the diluted nuclei, and intermolecular ligation was executed for 4 h at 16°C and then for 30 min at room temperature. DNA was purified using phenol-chloroform after decross-linking. Chromosome interaction was quantified by TaqMan qPCR, and BAC clone RP203-207L17 was used as a control. PCR products were examined on a 6% TBE-PAGE gel and TA cloned for Sanger sequencing to confirm the specific ligation. Primer and probe sequences used for 3C are listed in Supplemental Table S1.

### Luciferase reporter assay

BAT cells grown in a 96 well plate were transfected with 50 ng or a relatively equal molar amount of the indicated luciferase reporter based on the plasmid size and 0.2 ng of Renilla luciferase reporter using Lipofectamine 3000. Cells were differentiated 24 h after transfection and assayed for luciferase activity at day 5 of differentiation with or without 4 h of isoproterenol treatment using a dual-luciferase kit (Promega E1960) according to the manufacturer-recommended protocol. For the luciferase reporter cotransfected with *Etv4*, *Etv5*, or empty vector, 293FT cells were used and assayed 48 h after transfection.

### CRISPR deletion

Two PX458-gRNA or PX458-gScr (Addgene 48138) constructs were cotransfected into primary brown preadipocytes. GFP-positive cells indicating successful transfection of gRNA were sorted by FACS at 48 h after transfection. Sorted cells were cultured in a 24 well plate until 100% confluency and then differentiated for 5 days. Cells were collected for RT-qPCR and genome DNA. Genome DNAs were used for PCR with primers flanking the two gRNAs to amplify the edited region, and PCR products were TA cloned followed by Sanger sequencing. gRNA sequences used for CRISPR are listed in Supplemental Table S2, and RT-qPCR primer sequences are listed in Supplemental Table S3.

### Recombinant AAV vectors and tail vein injection

For CRISPRi, an AAV expression cassette was obtained by cloning between the ITRs of AAV2: a dSaCas9-KRAB under the control of a 221 bp *Ucp1* minimal promoter, with the addition of four tandem repeats of miR-122a target sequence that were in the 3′ untranslated region of the expression cassette. For CRISPRa, dSaCas9-VP64 under the control of a 577 bp *Fabp4* promoter was used. For gRNAs expression AAV cassette, human U6 promoter-driven scramble gRNA or gRNA targeting on −12 kb enhancer or on *Etv4* promoter with sacas9 binding scaffold was used. For Etv4 overexpression of AAV, the *Etv4* gene under the control of human *AP2.2* or the *Fabp4* promoter was used with the additional miR-122a target sequence. Single-strand AAV virus of serotype 8 was produced by cotransfecting AAV vector with pAAV2/8 and pHelper to AAVPro 293T cells. The AAV particle was purified using the AAVpro purification kit (Takara 632273). Eight week old C57BL/6J male mice were randomized and then i.v. injected with 1× 10^12^ genome copies per kilogram of body weight. The dCas9 AAV virus and gRNA AAV virus were mixed at equal amounts and injected through the tail vein. Phenotypes were measured at the indicated time points.

### CRISPRi and CRISPRa in vitro

For CRISPRi and CRISPRa in cell culture, BAT cells or human primary preadipocytes were transduced with lentivirus expressing dCas9-KRAB (Addgene 122205) or dcas9-VP64 (by replacing KRAB with VP64). Transduced cells were selected by 5 µg/mL blasticidin for 5 days to get cell line stably expressing dCas9-KRAB or dCas9-VP64. Stable cell lines were transduced with gRNA lentivirus (backbone from VectorBuilder) and sorted by FACS for GFP marker. Sorted cells were then differentiated for 5 days and collected for RNA and qPCR. gRNA sequences used for CRISPR are listed in Supplemental Table S2.

### Chromatin immunoprecipitation-qPCR

Differentiated BAT cells were cross-linked in 1% formaldehyde in PBS for 10 min. The manufacturer-recommended protocol from the SimpleChIP Plus sonication chromatin IP kit (Cell Signaling Technology 56383) was followed. Nuclei were isolated and sonicated on an M220 for 8 min at the manufacturer-recommended power settings. Soluble chromatin was quantified by absorbance at 260 nm, and 30 µg of chromatin was immunoprecipitated using 3 µg of the indicated antibodies or IgG control using buffers. Samples were analyzed by qPCR using the primer sets in Supplemental Table S3 for enrichment in the target region.

### Coimmunoprecipitation

AAV carrying HA-tagged Etv4 under the adiponectin promoter was directly injected into mouse brown adipose tissues at a dose of 1 × 10^10^ genome copies in 50 µL of PBS. Two weeks later, the tissue was lysed in cell lysis buffer with proteinase inhibitor, incubated with IgG or HA antibody (Cell Signaling Technology 3724) for 2 h, and captured by protein A/G beads. Beads were washed three times with PBS to remove the nonspecific binding and then eluted for subsequent immunoblotting using antibodies targeting the indicated proteins (anti-Etv4 [MyBioSource MBS9125463], anti-PPARg [Abcamab45036], anti-EBF2 [R&D AF7006], and anti-C/EBPb [Bethyl Laboratories A302-738A]).

### ATAC-seq

ATAC-seq was performed according to Corces et al. (2017). BAT and iWAT were homogenized by dounce homogenization in RSB buffer with 0.1% NP40, 0.01% digitonin, and 0.1% Tween-20. Homogenized tissue was incubated for 5 min on ice and then filtered into collection tubes through a 40 µm cell strainer. The collection was spun at 500*g* for 10 min at 4°C for nucleus precipitation. Nucleic pellets were resuspended in RSB buffer with 0.1% Tween-20 and counted under a microscope. Fifty-thousand nucleic pellets were aliquoted and spun at 500*g* for 5 min at 4°C. Nucleic pellets were resuspended in transposition mix (25 µL of 2× TD buffer, 2.5 µL of transposase [100 nM final], 16.5 of µL PBS, 0.5 µL of 1% digitonin, 0.5 µL of 10% Tween-20, 5 µL of H_2_O) and incubated for 30 min at 37°C. The reactions were then purified using Zymo DNA Clean and Concentrator-5 kit. The purified DNA was subjected to library preparation. Two biological replicates were generated for each condition. The libraries were sequenced with a HiSeq4000 Instrument (Illumina), and 150 PE reads were generated.

### CUT&RUN

CUT&RUN was performed using CUT&RUN Pro set (Antibodies-online ABIN6923138) following the published protocol (Skene and Henikoff 2017). BAT and iWAT were homogenized in NE buffer (20 mM HEPES-KOH at pH 7.9, 10 mM KCl, 1 mM MgCl_2_, 0.1% Triton X-100, 20% glycerol) and incubated for 5 min on ice. The lysis was filtered through a 40 µm cell strainer, and the flowthrough was spun at 500*g* for 5 min at 4°C. Nuclei were resuspended in 1 mL of buffer 1 (20 mM HEPES at pH 7.5, 150 mM NaCl, 2 mM EDTA, 0.5 mM spermidine, 0.1% BSA) and counted under a microscope. Five-hundred-thousand nucleic pellets were aliquoted and then added with 10 µL of CUT&RUN concanavalin A beads. Next, the nucleic pellets were washed in 1.5 mL of buffer 2 (20 mM HEPES at pH 7.5, 150 mM NaCl, 0.5 mM spermidine, 0.1% BSA). Nuclei were resuspended in 500 µL of buffer 2, and 1 µg of antibody (anti-H3k27ac [EpiCypher 13-0059] and anti-H3k27me3 [EpiCypher 13-0055]) was added and incubated for 2 h at 4°C. Nuclei were washed three times in 1 mL of buffer 2 to remove unbound antibody. Nuclei were resuspended in 300 mL of buffer 2, and 5 µL of pA-MN (Antibodies-online ABIN6950951) was added and incubated for 1 h at 4°C. Nuclei were washed three times in 0.5 mL of buffer 2 to remove unbound pA-MN. Nucleic pellets were resuspended in 300 µL of buffer 2 and incubated on ice. CaCl_2_ was quickly added to a final concentration of 2 mM, mixed well, and incubated for 15 min on ice. EDTA was added to 10 mM and EGTA was added to 20 mM to stop the reaction. The reaction was incubated for 30 min at 37°C. The supernatant was transferred and DNA was purified. CUT&RUN library preparation was performed following the KAPA Hyper preparation kit (KK8504).

### Analysis of CUT&RUN and ATAC-seq data

Data were aligned to mouse genome mm10 using Bowtie2 (Langmead and Salzberg 2012; Langmead et al. 2019). PCR duplicates and low-quality reads were removed by Picard (https://broadinstitute.github.io/picard). Aligned reads were processed by SAMtools (Danecek et al. 2021). Bigwig files were generated using BamCoverage in deepTools2 (Ramírez et al. 2016). Tracks were visualized by the WashU Epigenome Browser (Li et al. 2022).

### RNA-seq and analysis

RNA from BAT cells was purified using an RNeasy kit and RNase-free DNase set. RNA libraries were generated using a KAPA RNA HyperPrep kit (KK8561). The library was sequenced on a HiSeq4000 platform, generating 150 bp PE reads. Reads were aligned to mouse genome mm10 using STAR (Dobin et al. 2013). PCR duplicates and low-quality reads were removed by Picard (https://broadinstitute.github.io/picard). Filtered reads were assigned to the annotated transcriptome and quantified using featureCounts (Liao et al. 2014). Normalization and differential expression analysis were performed using edgeR (Robinson et al. 2010).

### Lentiviral particle packaging and transduction

Lentiviral gRNAs (sequences in Supplemental Table S2) targeting the −12 kb enhancer or *Etv4* promoter or scramble gRNA control plasmids were constructed by inserting the gRNA expression cassette into a lentivector with eGFP. shRNAs targeting *Etv4* (TRCN0000295466 and TRCN0000306806) and scramble controls (SHC002) were purchased from Sigma. Lentiviral plasmids were cotransfected with packaging plasmids psPAX2 and pMD2.G (obtained from Addgene as a gift from Didier Trono) into 293FT cells. Lentiviruses were harvested 48 and 72 h after transfection. Differentiated BAT cells were transduced at day 5 by shRNA lentivirus particle overnight, and cells were collected at day 6 for experiments.

### Metabolic and thermogenic measurement

Fat and lean mass were determined by echoMRI-100V. Oxygen consumption rate was measured using the comprehensive laboratory animal monitoring system (CLAMS). Data were normalized to lean body mass. Mice were individually caged and maintained under a 12 h light/12 h dark cycle. Body temperatures were assessed using a RET-3 rectal probe for mice (Physitemp). An FLIR E5 series infrared camera was used to capture thermography.

### Glucose tolerance and insulin sensitivity

Animals were fasted for 6 h with free access to drinking water. A baseline blood sample was collected from the tails, followed by an i.p. injection of glucose (2 g/kg body weight) for GTT or of insulin (0.75 U/kg body weight) for ITT, and blood was taken from the tails at the indicated time points after injection.

### Fasting insulin measurement

Animals were fasted for 6 h with free access to drinking water. Blood samples were collected from the tails into Microvette blood collection tubes. Blood samples were spun at 3000*g* for 3 min. Plasma samples were transferred into new tubes and then applied to ELISA using an ultrasensitive mouse insulin ELISA kit (Crystal Chem 90080) based on the manufacturer's instruction. Standard curves were fitted with a linear model.

### Statistics and reproducibility

All in vitro data were obtained from at least three independent replicate experiments. Graphs, calculations, and statistical analyses were performed using GraphPad Prism software. Data are shown as means ± SEM. Student's *t*-test or one-way/two-way ANOVA was used to compare the differences between two or more groups. Significance tests are indicated in the legends of the corresponding figure panels.

### Data availability

All sequencing data created in this study have been deposited at GEO at GSE243845.

## Supplemental Material

Supplement 1

## References

[GAD352748XUEC1] Boyer BB, Kozak LP. 1991. The mitochondrial uncoupling protein gene in brown fat: correlation between DNase I hypersensitivity and expression in transgenic mice. Mol Cell Biol 11: 4147–4156. 10.1128/mcb.11.8.4147-4156.1991 1712903 PMC361232

[GAD352748XUEC2] Cannon B, Nedergaard J. 2004. Brown adipose tissue: function and physiological significance. Physiol Rev 84: 277–359. 10.1152/physrev.00015.2003 14715917

[GAD352748XUEC3] Cao W, Daniel KW, Robidoux J, Puigserver P, Medvedev AV, Bai X, Floering LM, Spiegelman BM, Collins S. 2004. P38 mitogen-activated protein kinase is the central regulator of cyclic AMP-dependent transcription of the brown fat uncoupling protein 1 gene. Mol Cell Biol 24: 3057–3067. 10.1128/MCB.24.7.3057-3067.2004 15024092 PMC371122

[GAD352748XUEC4] Cassard-Doulcier AM, Gelly C, Bouillaud F, Ricquier D. 1998. A 211-bp enhancer of the rat uncoupling protein-1 (UCP-1) gene controls specific and regulated expression in brown adipose tissue. Biochem J 333: 243–246. 10.1042/bj3330243 9657961 PMC1219578

[GAD352748XUEC5] Chella Krishnan K, Vergnes L, Acín-Pérez R, Stiles L, Shum M, Ma L, Mouisel E, Pan C, Moore TM, Péterfy M, 2021. Sex-specific genetic regulation of adipose mitochondria and metabolic syndrome by Ndufv2. Nat Metab 3: 1552–1568. 10.1038/s42255-021-00481-w 34697471 PMC8909918

[GAD352748XUEC6] Cong L, Zhang F. 2015. Genome engineering using CRISPR–Cas9 system. Methods Mol Biol 1239: 197–217. 10.1007/978-1-4939-1862-1_10 25408407

[GAD352748XUEC7] Corces MR, Trevino AE, Hamilton EG, Greenside PG, Sinnott-Armstrong NA, Vesuna S, Satpathy AT, Rubin AJ, Montine KS, Wu B, 2017. An improved ATAC-seq protocol reduces background and enables interrogation of frozen tissues. Nat Methods 14: 959–962. 10.1038/nmeth.4396 28846090 PMC5623106

[GAD352748XUEC8] Cui X, Cao Q, Li F, Jing J, Liu Z, Yang X, Schwartz GJ, Yu L, Shi H, Shi H, 2024. The histone methyltransferase SUV420H2 regulates brown and beige adipocyte thermogenesis. JCI Insight 9: e164771. 10.1172/jci.insight.164771 38713533 PMC11382888

[GAD352748XUEC9] Cypess AM, Lehman S, Williams G, Tal I, Rodman D, Goldfine AB, Kuo FC, Palmer EL, Tseng YH, Doria A, 2009. Identification and importance of brown adipose tissue in adult humans. N Engl J Med 360: 1509–1517. 10.1056/NEJMoa0810780 19357406 PMC2859951

[GAD352748XUEC10] Danecek P, Bonfield JK, Liddle J, Marshall J, Ohan V, Pollard MO, Whitwham A, Keane T, McCarthy SA, Davies RM, 2021. Twelve years of SAMtools and BCFtools. Gigascience 10: giab008. 10.1093/gigascience/giab008 33590861 PMC7931819

[GAD352748XUEC11] Dempersmier J, Sambeat A, Gulyaeva O, Paul SM, Hudak CS, Raposo HF, Kwan HY, Kang C, Wong RH, Sul HS. 2015. Cold-inducible Zfp516 activates UCP1 transcription to promote browning of white fat and development of brown fat. Mol Cell 57: 235–246. 10.1016/j.molcel.2014.12.005 25578880 PMC4304950

[GAD352748XUEC12] Dobin A, Davis CA, Schlesinger F, Drenkow J, Zaleski C, Jha S, Batut P, Chaisson M, Gingeras TR. 2013. STAR: ultrafast universal RNA-seq aligner. Bioinformatics 29: 15–21. 10.1093/bioinformatics/bts635 23104886 PMC3530905

[GAD352748XUEC13] Emmett MJ, Lim HW, Jager J, Richter HJ, Adlanmerini M, Peed LC, Briggs ER, Steger DJ, Ma T, Sims CA, 2017. Histone deacetylase 3 prepares brown adipose tissue for acute thermogenic challenge. Nature 546: 544–548. 10.1038/nature22819 28614293 PMC5826652

[GAD352748XUEC14] Field A, Adelman K. 2020. Evaluating enhancer function and transcription. Annu Rev Biochem 89: 213–234. 10.1146/annurev-biochem-011420-095916 32197056

[GAD352748XUEC15] Furuhashi M, Hotamisligil GS. 2008. Fatty acid-binding proteins: role in metabolic diseases and potential as drug targets. Nat Rev Drug Discov 7: 489–503. 10.1038/nrd2589 18511927 PMC2821027

[GAD352748XUEC16] Galmozzi A, Sonne SB, Altshuler-Keylin S, Hasegawa Y, Shinoda K, Luijten IHN, Chang JW, Sharp LZ, Cravatt BF, Saez E, 2014. Thermomouse: an in vivo model to identify modulators of UCP1 expression in brown adipose tissue. Cell Rep 9: 1584–1593. 10.1016/j.celrep.2014.10.066 25466254 PMC4268417

[GAD352748XUEC17] Hagège H, Klous P, Braem C, Splinter E, Dekker J, Cathala G, de Laat W, Fornè T. 2007. Quantitative analysis of chromosome conformation capture assays (3C-qPCR). Nat Protoc 2: 1722–1733. 10.1038/nprot.2007.243 17641637

[GAD352748XUEC18] Harms MJ, Lim HW, Ho Y, Shapira SN, Ishibashi J, Rajakumari S, Steger DJ, Lazar MA, Won KJ, Seale P. 2015. PRDM16 binds MED1 and controls chromatin architecture to determine a brown fat transcriptional program. Genes Dev 29: 298–307. 10.1101/gad.252734.114 25644604 PMC4318146

[GAD352748XUEC19] Heinz S, Romanoski CE, Benner C, Glass CK. 2015. The selection and function of cell type-specific enhancers. Nat Rev Mol Cell Biol 16: 144–154. 10.1038/nrm3949 25650801 PMC4517609

[GAD352748XUEC20] Henriques T, Scruggs BS, Inouye MO, Muse GW, Williams LH, Burkholder AB, Lavender CA, Fargo DC, Adelman K. 2018. Widespread transcriptional pausing and elongation control at enhancers. Genes Dev 32: 26–41. 10.1101/gad.309351.117 29378787 PMC5828392

[GAD352748XUEC21] Inoue SI, Emmett MJ, Lim HW, Midha M, Richter HJ, Celwyn IJ, Mehmood R, Chondronikola M, Klein S, Hauck AK, 2024. Short-term cold exposure induces persistent epigenomic memory in brown fat. Cell Metab 36: 1764–1778.e9. 10.1016/j.cmet.2024.05.011 38889724 PMC11305953

[GAD352748XUEC22] Jang Y, Park YK, Lee JE, Wan D, Tran N, Gavrilova O, Ge K. 2021. MED1 is a lipogenesis coactivator required for postnatal adipose expansion. Genes Dev 35: 713–728. 10.1101/gad.347583.120 33888555 PMC8091974

[GAD352748XUEC23] Jimenez V, Muñoz S, Casana E, Mallol C, Elias I, Jambrina C, Ribera A, Ferre T, Franckhauser S, Bosch F. 2013. In vivo adeno-associated viral vector-mediated genetic engineering of white and brown adipose tissue in adult mice. Diabetes 62: 4012–4022. 10.2337/db13-0311 24043756 PMC3837045

[GAD352748XUEC24] Kajimura S, Seale P, Tomaru T, Erdjument-Bromage H, Cooper MP, Ruas JL, Chin S, Tempst P, Lazar MA, Spiegelman BM. 2008. Regulation of the brown and white fat gene programs through a PRDM16/CtBP transcriptional complex. Genes Dev 22: 1397–1409. 10.1101/gad.1666108 18483224 PMC2377193

[GAD352748XUEC25] Kim S, Wysocka J. 2023. Deciphering the multi-scale, quantitative *cis*-regulatory code. Mol Cell 83: 373–392. 10.1016/j.molcel.2022.12.032 36693380 PMC9898153

[GAD352748XUEC26] Koyano-Nakagawa N, Gong W, Das S, Theisen JWM, Swanholm TB, Van Ly D, Dsouza N, Singh BN, Kawakami H, Young S, 2022. Etv2 regulates enhancer chromatin status to initiate Shh expression in the limb bud. Nat Commun 13: 4221. 10.1038/s41467-022-31848-6 35864091 PMC9304341

[GAD352748XUEC27] Kozak UC, Kopecky J, Teisinger J, Enerbäck S, Boyer B, Kozak LP. 1994. An upstream enhancer regulating brown-fat-specific expression of the mitochondrial uncoupling protein gene. Mol Cell Biol 14: 59–67. 10.1128/mcb.14.1.59-67.1994 8264627 PMC358356

[GAD352748XUEC28] Lai B, Lee JE, Jang Y, Wang L, Peng W, Ge K. 2017. MLL3/MLL4 are required for CBP/p300 binding on enhancers and super-enhancer formation in brown adipogenesis. Nucleic Acids Res 45: 6388–6403. 10.1093/nar/gkx234 28398509 PMC5499743

[GAD352748XUEC29] Langmead B, Salzberg SL. 2012. Fast gapped-read alignment with Bowtie 2. Nat Methods 9: 357–359. 10.1038/nmeth.1923 22388286 PMC3322381

[GAD352748XUEC30] Langmead B, Wilks C, Antonescu V, Charles R. 2019. Scaling read aligners to hundreds of threads on general-purpose processors. Bioinformatics 35: 421–432. 10.1093/bioinformatics/bty648 30020410 PMC6361242

[GAD352748XUEC31] Li W, Notani D, Ma Q, Tanasa B, Nunez E, Chen AY, Merkurjev D, Zhang J, Ohgi K, Song X, 2013. Functional roles of enhancer RNAs for oestrogen-dependent transcriptional activation. Nature 498: 516–520. 10.1038/nature12210 23728302 PMC3718886

[GAD352748XUEC32] Li D, Purushotham D, Harrison JK, Hsu S, Zhuo X, Fan C, Liu S, Xu V, Chen S, Xu J, 2022. Washu Epigenome Browser update 2022. Nucleic Acids Res 50: W774–W781. 10.1093/nar/gkac238 35412637 PMC9252771

[GAD352748XUEC33] Liao Y, Smyth GK, Shi W. 2014. featureCounts: an efficient general purpose program for assigning sequence reads to genomic features. Bioinformatics 30: 923–930. 10.1093/bioinformatics/btt656 24227677

[GAD352748XUEC34] MacDougald OA, Cornelius P, Liu R, Lane MD. 1995. Insulin regulates transcription of the CCAAT/enhancer binding protein (C/EBP) α, β, and δ genes in fully-differentiated 3T3-L1 adipocytes. J Biol Chem 270: 647–654. 10.1074/jbc.270.2.647 7822291

[GAD352748XUEC35] Mach P, Kos PI, Zhan Y, Cramard J, Gaudin S, Tünnermann J, Marchi E, Eglinger J, Zuin J, Kryzhanovska M, 2022. Cohesin and CTCF control the dynamics of chromosome folding. Nat Genet 54: 1907–1918. 10.1038/s41588-022-01232-7 36471076 PMC9729113

[GAD352748XUEC36] Mei S, Qin Q, Wu Q, Sun H, Zheng R, Zang C, Zhu M, Wu J, Shi X, Taing L, 2017. Cistrome Data Browser: a data portal for ChIP-seq and chromatin accessibility data in human and mouse. Nucleic Acids Res 45: D658–D662. 10.1093/nar/gkw983 27789702 PMC5210658

[GAD352748XUEC37] Mikhaylichenko O, Bondarenko V, Harnett D, Schor IE, Males M, Viales RR, Furlong EEM. 2018. The degree of enhancer or promoter activity is reflected by the levels and directionality of eRNA transcription. Genes Dev 32: 42–57. 10.1101/gad.308619.117 29378788 PMC5828394

[GAD352748XUEC38] Park KW, Waki H, Choi SP, Park KM, Tontonoz P. 2010. The small molecule phenamil is a modulator of adipocyte differentiation and PPARγ expression. J Lipid Res 51: 2775–2784. 10.1194/jlr.M008490 20519739 PMC2918460

[GAD352748XUEC39] Pham D, Moseley CE, Gao M, Savic D, Winstead CJ, Sun M, Kee BL, Myers RM, Weaver CT, Hatton RD. 2019. Batf pioneers the reorganization of chromatin in developing effector T cells via Ets1-dependent recruitment of Ctcf. Cell Rep 29: 1203–1220.e7. 10.1016/j.celrep.2019.09.064 31665634 PMC7182170

[GAD352748XUEC40] Qi LS, Larson MH, Gilbert LA, Doudna JA, Weissman JS, Arkin AP, Lim WA. 2013. Repurposing CRISPR as an RNA-guided platform for sequence-specific control of gene expression. Cell 152: 1173–1183. 10.1016/j.cell.2013.02.022 23452860 PMC3664290

[GAD352748XUEC41] Rajbhandari P, Thomas BJ, Feng AC, Hong C, Wang J, Vergnes L, Sallam T, Wang B, Sandhu J, Seldin MM, 2018. IL-10 signaling remodels adipose chromatin architecture to limit thermogenesis and energy expenditure. Cell 172: 218–233.e17. 10.1016/j.cell.2017.11.019 29249357 PMC5766418

[GAD352748XUEC42] Ramírez F, Ryan DP, Grüning B, Bhardwaj V, Kilpert F, Richter AS, Heyne S, Dündar F, Manke T. 2016. deepTools2: a next generation web server for deep-sequencing data analysis. Nucleic Acids Res 44: W160–W165. 10.1093/nar/gkw257 27079975 PMC4987876

[GAD352748XUEC43] Rim JS, Kozak LP. 2002. Regulatory motifs for CREB-binding protein and Nfe2l2 transcription factors in the upstream enhancer of the mitochondrial uncoupling protein 1 gene. J Biol Chem 277: 34589–34600. 10.1074/jbc.M108866200 12084707

[GAD352748XUEC44] Robidoux J, Cao W, Quan H, Daniel KW, Moukdar F, Bai X, Floering LM, Collins S. 2005. Selective activation of mitogen-activated protein (MAP) kinase kinase 3 and p38α MAP kinase is essential for cyclic AMP-dependent UCP1 expression in adipocytes. Mol Cell Biol 25: 5466–5479. 10.1128/MCB.25.13.5466-5479.2005 15964803 PMC1157000

[GAD352748XUEC45] Robinson MD, McCarthy DJ, Smyth GK. 2010. edgeR: a Bioconductor package for differential expression analysis of digital gene expression data. Bioinformatics 26: 139–140. 10.1093/bioinformatics/btp616 19910308 PMC2796818

[GAD352748XUEC46] Sandoval GJ, Pulice JL, Pakula H, Schenone M, Takeda DY, Pop M, Boulay G, Williamson KE, McBride MJ, Pan J, 2018. Binding of TMPRSS2-ERG to BAF chromatin remodeling complexes mediates prostate oncogenesis. Mol Cell 71: 554–566.e7. 10.1016/j.molcel.2018.06.040 30078722 PMC6140332

[GAD352748XUEC47] Segawa K, Matsuda M, Fukuhara A, Morita K, Okuno Y, Komuro R, Shimomura I. 2009. Identification of a novel distal enhancer in human adiponectin gene. J Endocrinol 200: 107–116. 10.1677/JOE-08-0376 18931025

[GAD352748XUEC48] Shapira SN, Lim HW, Rajakumari S, Sakers AP, Ishibashi J, Harms MJ, Won KJ, Seale P. 2017. EBF2 transcriptionally regulates brown adipogenesis via the histone reader DPF3 and the BAF chromatin remodeling complex. Genes Dev 31: 660–673. 10.1101/gad.294405.116 28428261 PMC5411707

[GAD352748XUEC49] Shen Y, Su Y, Silva FJ, Weller AH, Sostre-Colón J, Titchenell PM, Steger DJ, Seale P, Soccio RE. 2020. Shared PPARα/γ target genes regulate brown adipocyte thermogenic function. Cell Rep 30: 3079–3091.e5. 10.1016/j.celrep.2020.02.032 32130908

[GAD352748XUEC50] Skene PJ, Henikoff S. 2017. An efficient targeted nuclease strategy for high-resolution mapping of DNA binding sites. eLife 6: e21856. 10.7554/eLife.21856 28079019 PMC5310842

[GAD352748XUEC51] Tontonoz P, Hu E, Graves RA, Budavari AI, Spiegelman BM. 1994. mPPARγ2: tissue-specific regulator of an adipocyte enhancer. Genes Dev 8: 1224–1234. 10.1101/gad.8.10.1224 7926726

[GAD352748XUEC52] van der Lans AA, Hoeks J, Brans B, Vijgen GH, Visser MG, Vosselman MJ, Hansen J, Jörgensen JA, Wu J, Mottaghy FM, 2013. Cold acclimation recruits human brown fat and increases nonshivering thermogenesis. J Clin Invest 123: 3395–3403. 10.1172/JCI68993 23867626 PMC3726172

[GAD352748XUEC53] Virtanen KA, Lidell ME, Orava J, Heglind M, Westergren R, Niemi T, Taittonen M, Laine J, Savisto NJ, Enerbäck S, 2009. Functional brown adipose tissue in healthy adults. N Engl J Med 360: 1518–1525. 10.1056/NEJMoa0808949 19357407

[GAD352748XUEC54] Yi D, Dempersmier JM, Nguyen HP, Viscarra JA, Dinh J, Tabuchi C, Wang Y, Sul HS. 2019. Zc3h10 acts as a transcription factor and is phosphorylated to activate the thermogenic program. Cell Rep 29: 2621–2633.e4. 10.1016/j.celrep.2019.10.099 31775033 PMC6911170

[GAD352748XUEC55] Yi D, Nguyen HP, Dinh J, Viscarra JA, Xie Y, Lin F, Zhu M, Dempersmier JM, Wang Y, Sul HS. 2020. Dot1l interacts with Zc3h10 to activate Ucp1 and other thermogenic genes. eLife 9: e59990. 10.7554/eLife.59990 33107819 PMC7661038

[GAD352748XUEC56] Yoneshiro T, Wang Q, Tajima K, Matsushita M, Maki H, Igarashi K, Dai Z, White PJ, McGarrah RW, Ilkayeva OR, 2019. BCAA catabolism in brown fat controls energy homeostasis through SLC25A44. Nature 572: 614–619. 10.1038/s41586-019-1503-x 31435015 PMC6715529

[GAD352748XUEC57] Zheng R, Wan C, Mei S, Qin Q, Wu Q, Sun H, Chen CH, Brown M, Zhang X, Meyer CA, 2019. Cistrome Data Browser: expanded datasets and new tools for gene regulatory analysis. Nucleic Acids Res 47: D729–D735. 10.1093/nar/gky1094 30462313 PMC6324081

