# Supplementary material for: A distal enhancer with ETV4 binding is critical for UCP1 expression and thermogenesis in brown fat
Source: Genes Dev. 2025 Jul 1;39(13-14):808–25. doi: 10.1101/gad.352748.125 (PMC12211996; doi:10.1101/gad.352748.125)
Supplement: Supplement 1 [file Supplemental_Data.pdf]

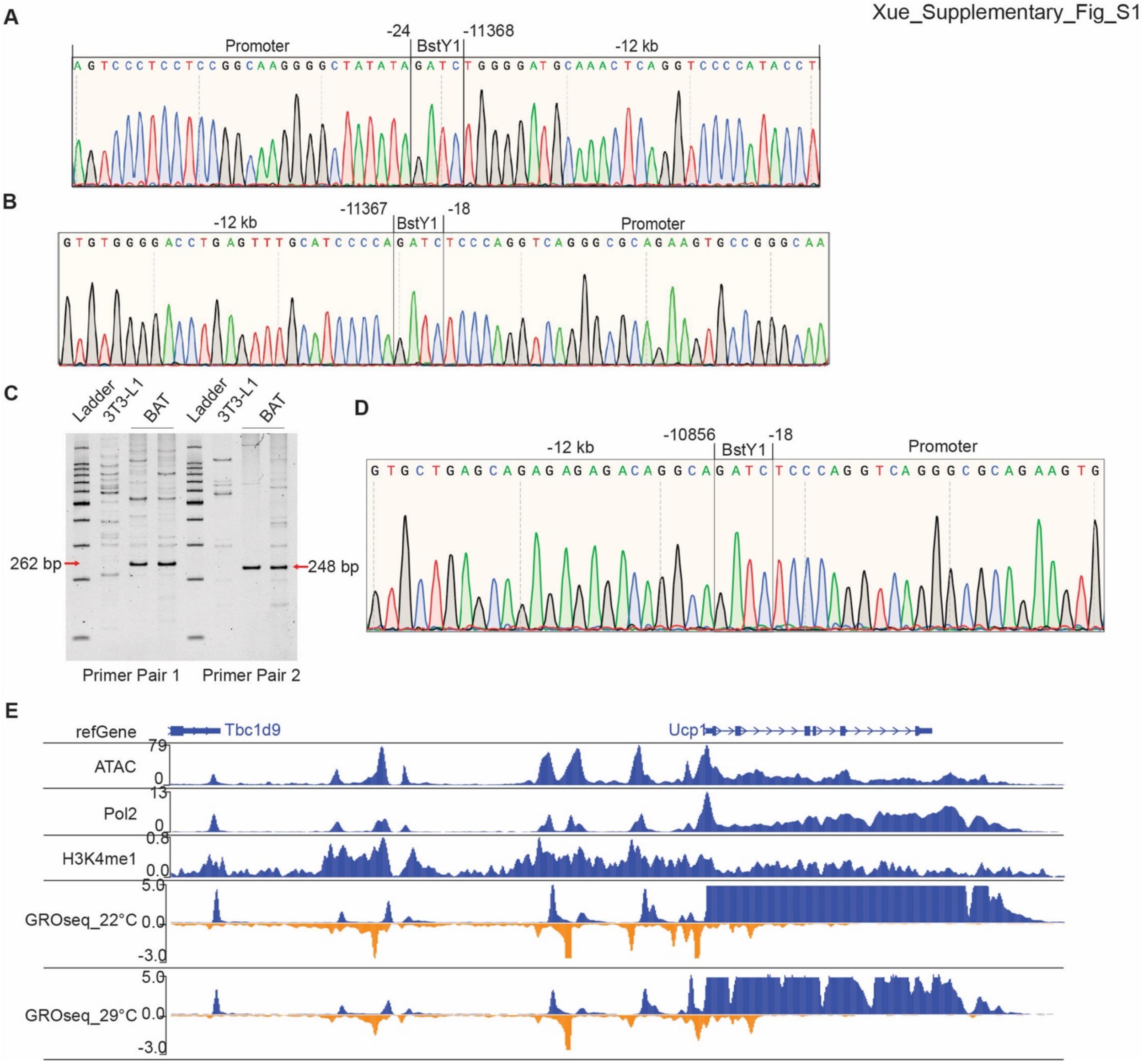

Figure S1. Interaction between *Ucp1* -12 kb enhancer with *Ucp1* proximal promoter. (A) Sanger sequencing to confirm the 3C product in brown adipose tissue. (B) Sanger sequencing to confirm the 3C product in differentiated BAT cells. (C) Visualization of the 3C PCR products amplified using two different primer pairs in differentiated BAT and 3T3-L1 cells. (D) Sanger sequencing to confirm the 3C product amplified by primer pair 2 in BAT cells. (E) Genome browser of RNA Pol2, H3K4me1 ChIP-seq, and GRO-seq on Ucp1 genome locus.

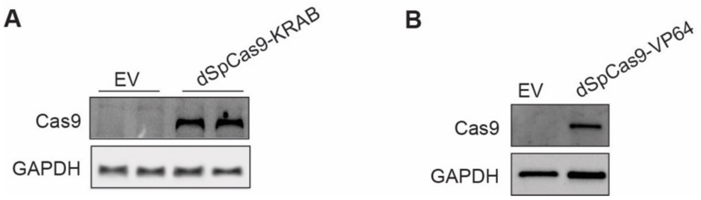

Figure S2. Stable expression of dCas9-KRAB and dCas9-VP64 in BAT cells. Immunoblotting to confirm the over expression of dCas9-KRAB (A) and dCas9-VP64 (B) in BAT cells.

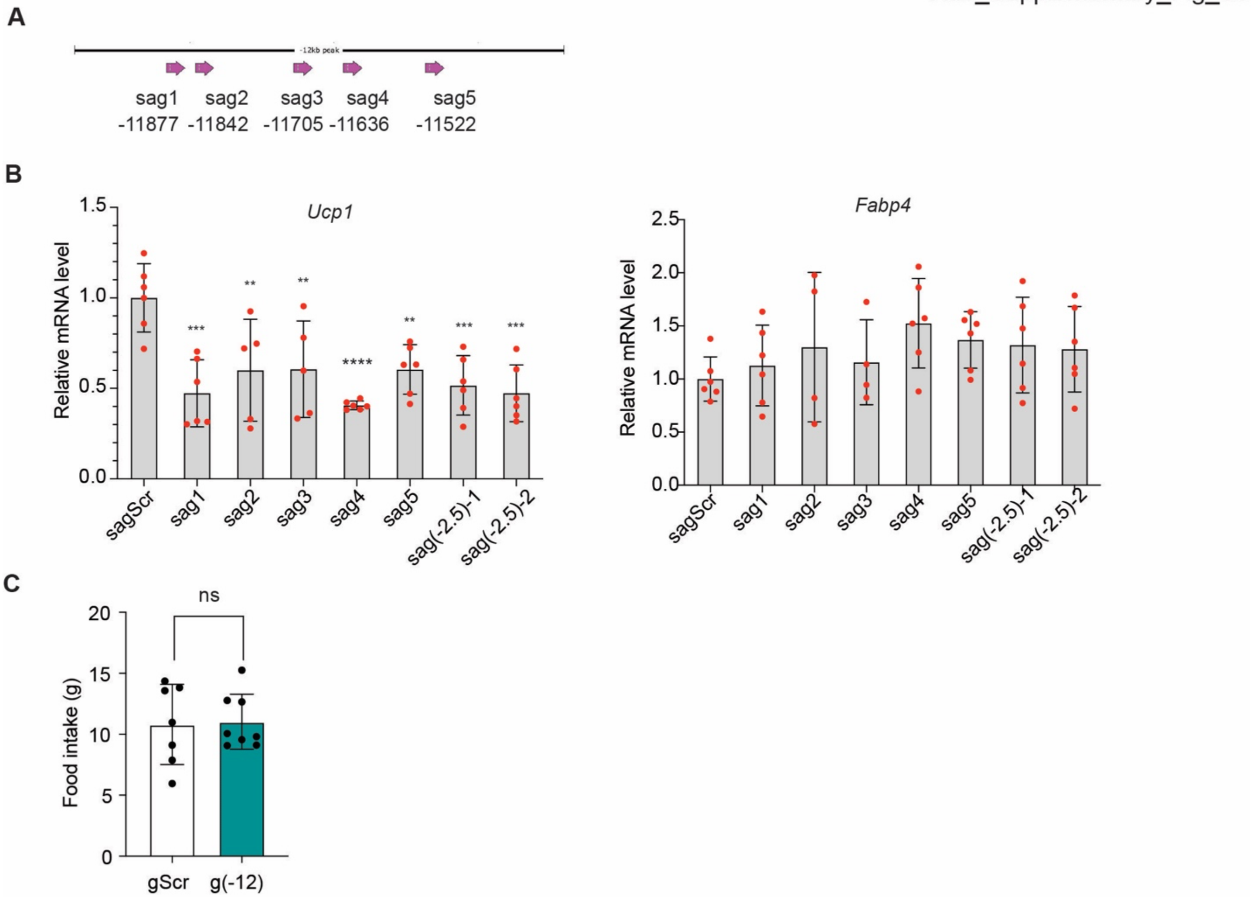

Figure S3. Repression of the -12kb enhancer suppresses thermogenesis in mice. (A) sagRNA targeting -12kb enhancer designed for AAV mediated CRISPRi. (B) *Ucp1* (left) and *Fabp4* (right) RNA level in differentiated CRISPRi BAT cells. (C) Food intake of control and CRISPRi mice. ** $p< 0.01$; *** $p< 0.005$; **** $p<0.001$; ns: non-significant based on one-way ANOVA (B) and Student's t-test (C). All error bars represent the mean ± SEM.

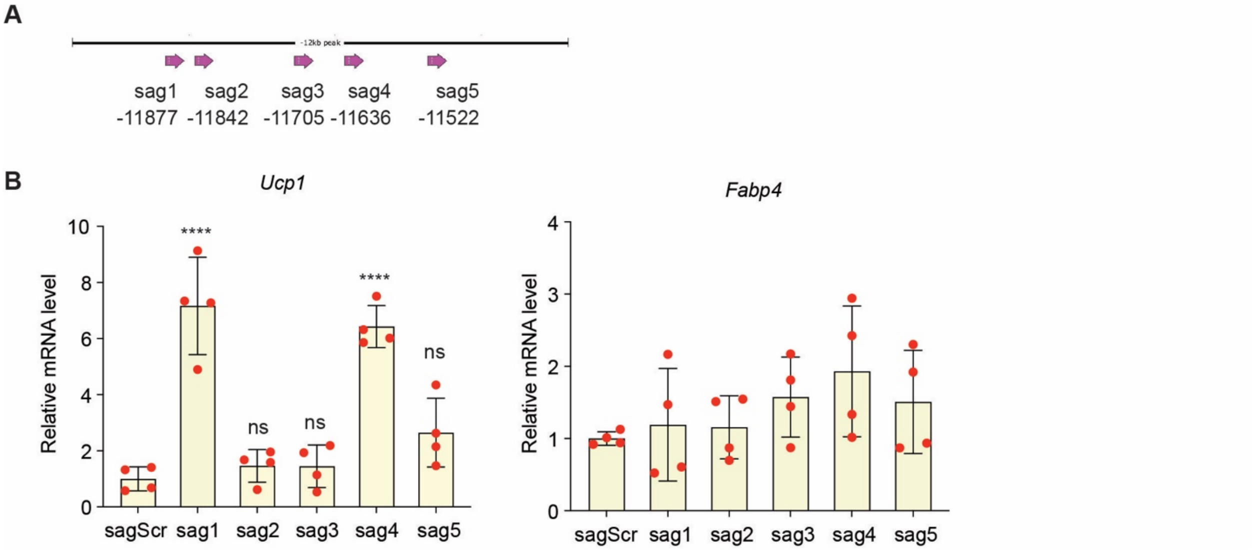

Figure S4. Activation of Ucp1 -12 kb enhancer by CRISPRa promotes thermogenesis. (A) sagRNA targeting -12kb enhancer designed for AAV mediated CRISPRa. (B) *Ucp1* (left) and *Fabp4* (right) RNA levels in CRISPRa brown adipocytes. **** $p<0.001$; ns: non-significant based on one-way ANOVA (B). All error bars represent the mean ± SEM.

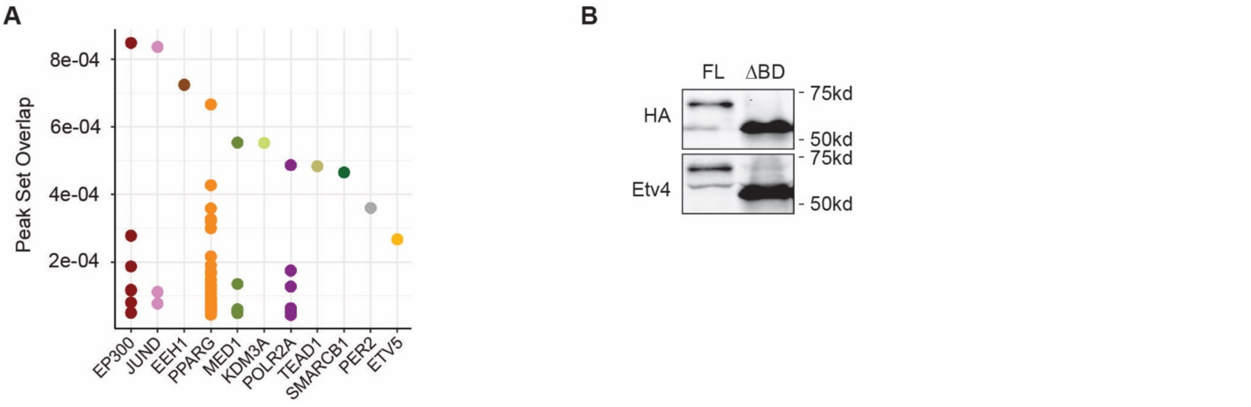

Figure S5. Etv4 activates Ucp1 by binding at -12 kb enhancer and promotes chromatin accessibility. (A) Peak Set Overlap analysis of publicly available ChIP-seq data showing the binding of indicated chromatin regulators and transcription factors on -12 kb Ucp1 enhancer. (B) Immunoblotting of full-length (FL) and DNA-binding domain deletion (ΔBD) Etv4.

Xue_Supplementary_Fig_S6

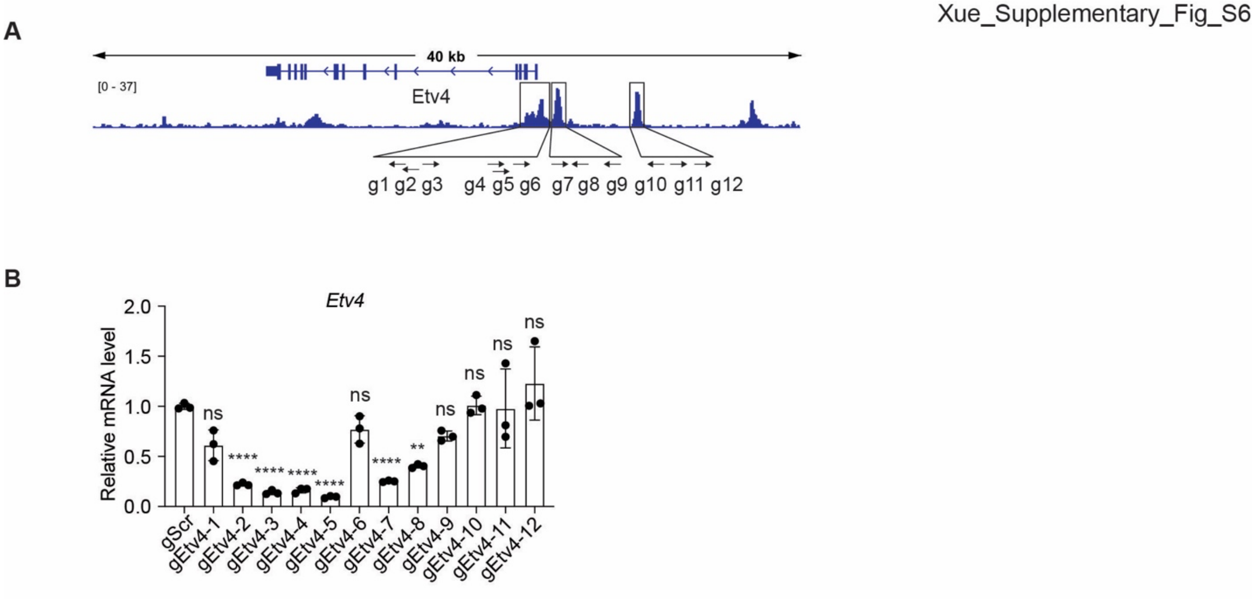

Figure S6. Repression of Etv4 inhibits thermogenesis in mice. (A) gRNAs targeting at the *Etv4* promoter were used for testing CRISPRi efficiency. (B) *Etv4* RNA levels in *Etv4* CRISPRi brown adipocytes. ** p<0.01; **** p<0.001; ns: non-significant based on one-way ANOVA (B). All error bars represent the mean ± SEM.

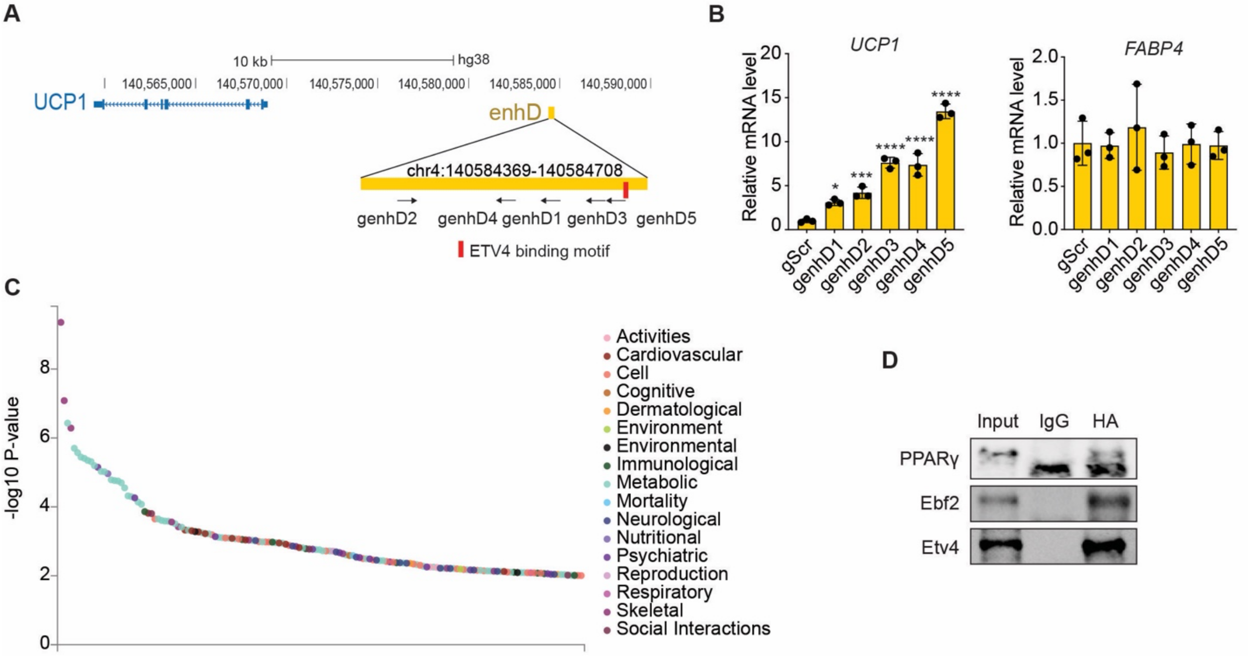

Figure S7. Related to Discussion. (A) Diagram of human Ucp1 and upstream genome locus. enhD: enhancer like domain; genhD: gRNA targets on enhD; Red bar indicate ETV4 binding motif. (B) RNA levels of *UCP1* and *FABP4* in human beige adipocytes. (C) Phenome-wide association study (PheWAS) of *Etv4*. (D) CoIP showing the interactions of Etv4 with PPARγ or Ebf2 in brown adipose tissue. * $p<0.05$; *** $p<0.005$; **** $p<0.001$; ns: non-significant based on one-way ANOVA (B). All error bars represent the mean ± SEM.

Supplementary Table 1. Primers and probes for 3C PCR and qPCR

	Description	sequence (5'-3')
F1	For 3C qPCR, paired with C	GGTGTTGACTGACTCAATTGC
F2	For 3C qPCR, paired with C	GTAGTGGGTTGGTCTGATGC
F3	For 3C qPCR, paired with C	CAAAAGCATAACCCAGGACGG
F4	For 3C qPCR, paired with C	CCACCTTCTCAGAAACTGAGTG
F5	For 3C qPCR, paired with C	AACTGAGTGGAGAGCCTAGT
F6	For 3C qPCR, paired with C	GGCTTTTAATGCCAGTGCTG
F7	For 3C qPCR, paired with C	ACAACCATCCATAATGAGATC
F8	For 3C qPCR, paired with C	GCAGTTCAAGATACATCCTG
C1	For 3C qPCR, paired with F	ACTAGCAGCTCTTTGGAGACC
Probe	For 3C qPCR	/56-FAM/CCCTTGCCG/ZEN/GAGGAGGGACTGG/3IABkFQ/
Primer pair1 (Forward)	For 3C PCR, with 262 bp PCR product	ACTAGCAGCTCTTTGGAGACC
Primer pair1 (Reverse)	For 3C PCR, with 262 bp PCR product	CAAAAGCATAACCCAGGACGG
Primer pair2 (Forward)	For 3C PCR, with 248 bp PCR product	GGCTTTTAATGCCAGTGCTG
Primer pair2 (Reverse)	For 3C PCR, with 248 bp PCR product	AGCCCTGAAGACAACAGTGG
Primer pair3 (Forward)	For 3C PCR, with 282 bp PCR product	ACTAGCAGCTCTTTGGAGACC
Primer pair3 (Reverse)	For 3C PCR, with 282 bp PCR product	CAAAAGCATAACCCAGGACGG

Supplementary Table 2. gRNA target sequences for CRISPR, CRISPRi and CRISPRa

	Description	target sequence with PAM (5'-3')
gScr	for CRISPR deletion, CRISPRi and CRISPRa	GCACTACCAGAGCTAACTCA
g1	for CRISPR deletion, CRISPRi and CRISPRa on -12 kb enhancer in cells compatible with SpCas9	GAAGAGGCTTGTGGTGCCTA
g2	for CRISPR deletion, CRISPRi and CRISPRa on -12 kb enhancer in cells compatible with SpCas9	CTGCCATCAAATACTTCCAC
g3	for CRISPRi and CRISPRa on -12 kb enhancer in cells compatible with SpCas9	TTACCGTGTAGCTTCCTCTC
g4	for CRISPRi and CRISPRa on -12 kb enhancer in cells compatible with SpCas9	GACTCAGTCAGACAATTCCC
g5	for CRISPRi and CRISPRa on -12 kb enhancer in cells compatible with SpCas9	CACGATTGCAGAATGTGTAG
g6	for CRISPRi and CRISPRa on -12 kb enhancer in cells compatible with SpCas9	AGGGCAAAGGTTGGCCGTCC
g7	for CRISPRi and CRISPRa on -12 kb enhancer in cells compatible with SpCas9	GGGTCCTCAAGTGCAATGCT
g8	for CRISPR deletion, CRISPRi and CRISPRa on -12 kb enhancer in cells compatible with SpCas9	AGCCTAGTAGACAGCGCTGT
g9	for CRISPR deletion, CRISPRi and CRISPRa on -12 kb enhancer in cells compatible with SpCas9	TTGCCAACAGCGCTGTCTACT
g(-2.5)	for CRISPRi on -2.5 kb region in cells compatible with SpCas9	GTGACCGGGTGCCCTGTAAA
sag1	for CRISPRi and CRISPRa on -12 kb enhancer in cell and mice compatible with SaCas9	AAGTATTTGATGGCAGGTAAC

sag2	for CRISPRi and CRISPRa on -12 kb enhancer in cell and mice compatible with SaCas9	GAGAGGAAGCTACACGGTAAA
sag3	for CRISPRi and CRISPRa on -12 kb enhancer in cell and mice compatible with SaCas9	TCCTGGCCAGCACACTGATGG
sag4	for CRISPRi and CRISPRa on -12 kb enhancer in cell and mice compatible with SaCas9	AGCCTAACACCGTGCTTCTCA
sag5	for CRISPRi and CRISPRa on -12 kb enhancer in cell and mice compatible with SaCas9	GTGCAATGCTCGGAGTGAGCT
gEtv4-1	for CRISPRi on Etv4 in cell and mice compatible with SaCas9	AGGTGGCGGGGCTAGGCCGGA
gEtv4-2	for CRISPRi on Etv4 in cell and mice compatible with SaCas9	CGGAGCGAGGCGGCCTGAGCC
gEtv4-3	for CRISPRi on Etv4 in cell and mice compatible with SaCas9	CTGCTGCGCCCGGAAAACAAG
gEtv4-4	for CRISPRi on Etv4 in cell and mice compatible with SaCas9	CAAGCTCAGCTAGCCGAGTCT
gEtv4-5	for CRISPRi on Etv4 in cell and mice compatible with SaCas9	TCCTCCCTCAAGCTCAGCTAG
gEtv4-6	for CRISPRi on Etv4 in cell and mice compatible with SaCas9	GAATGGAAGTCCAAAAAGTGA
gEtv4-7	for CRISPRi on Etv4 in cell and mice compatible with SaCas9	ATTTACTGAAGGCAGTGATGC
gEtv4-8	for CRISPRi on Etv4 in cell and mice compatible with SaCas9	TGTGAGAAAGACTACCTCCCT
gEtv4-9	for CRISPRi on Etv4 in cell and mice compatible with SaCas9	ATGCAATCTCTTCCAGCCTGT
gEtv4-10	for CRISPRi on Etv4 in cell and mice compatible with SaCas9	TGAAACACTGTCTCATGAGGA
gEtv4-11	for CRISPRi on Etv4 in cell and mice compatible with SaCas9	GTCACCAGCGCCTTCTAAAAA
gEtv4-12	for CRISPRi on Etv4 in cell and mice compatible with SaCas9	AAAAGAGCTTTGCGTGTGCAA
genhD1	for CRISPRa on human UCP1 enhD in cell compatible with SpCas9	GTGCATAACTAACTTGACTG
genhD2	for CRISPRa on human UCP1 enhD in cell compatible with SpCas9	AGAATCTCCCAATCTATCAG
genhD3	for CRISPRa on human UCP1 enhD in cell compatible with SpCas9	AGTGGGGAAGTGCAATAGAA
genhD4	for CRISPRa on human UCP1 enhD in cell compatible with SpCas9	AAAGTGTCTAACCACCCTGG
genhD5	for CRISPRa on human UCP1 enhD in cell compatible with SpCas9	CAGGGTAGGGAGGAATAGTT

Supplementary Table 3. Primers for RT-qPCR and ChIP-qPCR

	Forward (5'-3')	Reverse (5'-3')
18S	AGTCCCTGCCCTTTGTACACA	CGATCCGAGGGCCTCACTA
TBP	ACCCTTCACCAATGACTCCTATG	TGACTGCAGCAAATCGCTTGG
Ucp1	ACTGCCACACCTCCAGTCATT	CTTTGCCTCACTCAGGATTGG
Fabp4	ACACCGAGATTTCCT TCAAACTG	CCATCTAGGGTTATG ATGCTCTTCA
-12a eRNA	CGTGCACAAAAGCATAACCCA	GTGGGTCCTCAAGTGCAATG
-12b eRNA	CTCACTGAGAAGCACGGTGT	AAGTGCAATGCTCGGAGTGA
-2.5 eRNA	CAAAGCGCTGTGATGCTTT	GGTCACCATTTGCTCACTCA
-11 eRNA	GAACAAAGCCATAGAATGATGATGA	AAGGCCTACTCGATGTCCCT

Etv4	TTCTCTGGACCCTCTCCAGG	GCTCCAATCCCTTCCTGCTT
-12kb-P1	GCCAACCTTTGCCCTTTGAG	TTCTGAGAAGGTGGCTGCTG
-12kb-P2	CGTGCACAAAAGCATAACCCA	GTGGGTCCTCAAGTGCAATG
-12kb-P3	TTACCAGGAGGAGCCTGAGC	CCACAGAAGAGGCTTGTGGT
-12kb-P4	CTCACTGAGAAGCACGGTGT	AAGTGCAATGCTCGGAGTGA
actin	TATAAAACCCGGCGGCGCA	AGGAGCTGCAAAGAAGCTGT
